# Mechanical Characterization of Multilayered Hydrogels: A Rheological Study for 3D-Printed Systems

**DOI:** 10.1021/acs.biomac.1c00078

**Published:** 2021-03-18

**Authors:** Ana M. Fuentes-Caparrós, Zaloa Canales-Galarza, Michael Barrow, Bart Dietrich, Jörg Läuger, Markus Nemeth, Emily R. Draper, Dave J. Adams

**Affiliations:** † School of Chemistry, University of Glasgow, Glasgow G12 8QQ, U.K.; ‡ Department of Chemical Engineering, Faculty of Sciences, University of Granada, 18071 Granada, Spain; § Anton Paar Ltd., St. Albans AL4 0LA, U.K.; ∥ Anton Paar Germany, 73760 Ostfildern, Germany

## Abstract

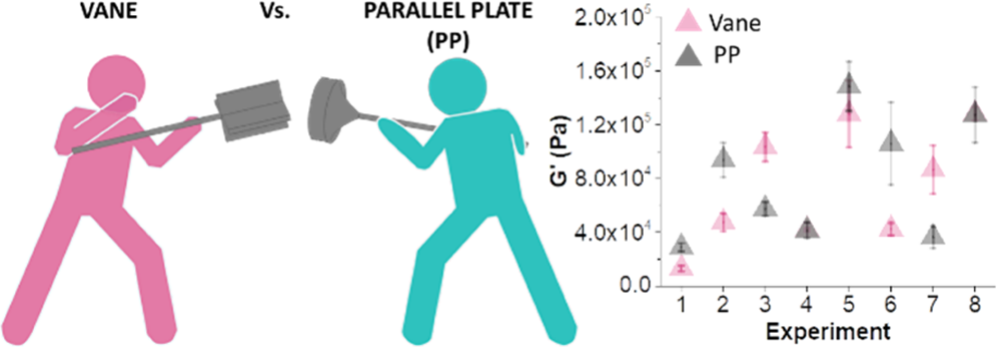

We describe rheological protocols to study layered and three-dimensional (3D)-printed gels. Our methods allow us to measure the properties at different depths and determine the contribution of each layer to the resulting combined properties of the gels. We show that there are differences when using different measuring systems for rheological measurement, which directly affects the resulting properties being measured. These methods allow us to measure the gel properties after printing, rather than having to rely on the assumption that there is no change in properties from a preprinted gel. We show that the rheological properties of fluorenylmethoxycarbonyl-diphenylalanine (FmocFF) gels are heavily influenced by the printing process.

## Introduction

Low-molecular-weight hydrogels (LMWGs) are formed by the self-assembly of small molecules into long anisotropic structures, mainly fibers, through noncovalent interactions.^1−4^ These fibers entangle and/or cross-link, immobilizing the solvent and forming a self-supporting three-dimensional (3D) network.^3^ Such hydrogels are mainly composed of water but still possess properties more reminiscent of a solid and can therefore exhibit both elastic and viscous responses.^5^ In fact, the viscoelastic nature of this class of material makes them potentially suitable for multiple biological applications such as tissue engineering, where materials capable of mimicking living tissues are needed.^6^ Furthermore, such hydrogels can encapsulate different types of molecules including proteins, growth factors, and signaling molecules that will facilitate cell proliferation and differentiation.^7^ Another key property is the ease of disrupting the interactions that hold together the molecules, making this class of materials responsive to a wide variety of external stimuli (for example, light, enzymes, or heat).^8−10^ As such, there is a significant interest in this class of materials, with much of the emphasis being on their applications in biomedical fields, such as controlled drug delivery, self-healing, and scaffolding for tissue engineering.^11−13^

Hydrogels can be used as cell-containing scaffolds for tissue engineering by delivering cells into damaged tissues and reconstructing organs in similar shapes. Strategies in the field of tissue engineering and regenerative medicine are committed to 3D scaffolds that mimic the natural extracellular matrix, which supports cell adhesion, migration, differentiation, and proliferation.^11^ One strategy requires seeding cells onto a 3D scaffold that supports in vitro tissue formation, which is then implanted into a patient for tissue repair. Intrinsically, the purpose of tissue engineering is to develop responsive living tissues with properties similar to those of living tissues that are intended to be replaced.

Typically, LMWGs are prepared as uniform systems with homogeneous properties.^21^ However, it is possible to make hierarchical hydrogels containing different layers with specific mechanical properties to mimic living-like tissues.^22^ Organs, for example, are spatially heterogeneous in terms of composition and, therefore, different cell types coexist within them. Consequently, multilayered hydrogels with different mechanical properties are of interest as an excellent option for 3D scaffold construction for tissue engineering.^23^ In recent years, a great deal of interest has been put into the fabrication of multilayered scaffold-based hydrogels for tissue engineering and regenerative medicine.^24−26^

Most recently, multiple technologies have been developed for the fabrication of hydrogels consisting of multiple layers including photolithography, microfluidics, and three-dimensional (3D) (bio)printing.^23^ 3D printing or additive manufacturing is a technology based on the computer-controlled layer-by-layer deposition of material (ink) that can create complex and well-defined three-dimensional objects with almost any shape or geometry.^27^ The 3D printing technology has revolutionized the biomedical field by providing a tool capable of manufacturing materials with unique control, flexibility, speed, and precision.

Extrusion-based 3D printing is useful for scaffold construction and has been used extensively for hydrogel printing over the last decade.^28,29^ Three-dimensional printers are widely used to print polymer gels for the fabrication of (bio)materials.^30−32^ However, due to the often poor mechanical properties that LMWGs possess and the relatively small number of gelators that can give gels with appropriate thixotropic characteristics, their use in extrusion-based 3D printing is not currently widespread. Nevertheless, interest in the use of 3D printing LMWGs is growing.^33−35^ The main considerations for a suitable ink are its printability, structural adhesion, and stability after printing. In terms of printability, shear-thinning and thixotropic hydrogels are ideal candidates as they can be easily extruded and they recover their original shape after the stress is released.^36,37^ Nolan et al. have previously reported the printability of some LMWGs using an extrusion-based printer and optimized the printing conditions.^33^ Gels formed from spherulitic domains of fibers exhibited better printability compared to gels formed from dense fibrous networks. The differences in printability rely on the fact that the gels with the underlying spherulitic domains are not as strongly affected by the shear process when being extruded through the nozzle. For the pH-triggered gels, the networks are strongly affected by the shear process, resulting in large-scale deformation. Hydrogels made using a solvent trigger, which results in the formation of spherulitic-like domains of fibers, are more suitable for 3D printing than pH-triggered gels, where a more uniform distribution of long fibers is formed that is more affected by the shear stress during extrusion.^33^ In general, for supramolecular gels formed via noncovalent interactions, the printability of the gel using an extrusion approach depends not only on the yield point of the gel but also on how well it recovers after being extruded, i.e., on its thixotropic nature.^38,39^

From a rheological point of view, control of the mechanical properties of printed hydrogels is crucial for the formation of an appropriate environment for cell growth, ensuring appropriate cellular functions. Shear rheometry is one of the most used techniques to define the mechanical properties of hydrogels. This technique allows us to characterize the rheological properties of the bulk material. Microrheology has also been used to measure the viscoelastic properties of soft materials in their local environment using local probe particles.^40^ Recently, Crosby and co-workers developed a new rheological method, cavitation rheology, which allows us to quantify the mechanical properties of soft materials in a local point with no need to add probe particles.^41^ This new technique has attracted the attention of many research groups that have used this new method to characterize different gel systems.^42−46^

Many studies have focused on the dynamic modification of the stiffness and elasticity of the hydrogels using different approaches as a means of tuning their physicochemical properties.^47^ It is common to find in the literature the suitability of polymer-based hydrogels for 3D printing by assessing their mechanical properties before printing, with little if any rheological characterization of the gels after they have been printed. As such, it is presumably assumed that the resulting mechanical properties of the printed materials are not affected by the printing process, which seems unlikely considering the process involved. This lack of measuring post printing is undoubtedly due to the difficulty in carrying out such measurements. In addition to demonstrating the ability of forming 3D-printed materials into complex shapes and structures, it is also necessary to evaluate the effect of the printing process on the mechanical properties of the resulting 3D-printed system. Numerous researchers have reported the ability to use 3D printing to fabricate polymer-based gel constructs.^30−32^ Where the mechanical properties are assessed for gels after printing, very few studies choose rheology as the main characterization technique. For example, Mondal et al. used sodium-gelatine hydrogels for 3D printing scaffolds. The stiffness of the resulting printed constructs was evaluated using rheology.^48^ More often, compression tests are used to characterize the mechanical properties of the printed constructs.^49−51^

To the best of our knowledge, changes in shear moduli of different patterned multilayered gels using rheology have not been reported in the literature. There have been examples where compression tests have been used to calculate the moduli. Hu and co-workers for example have tested the mechanical properties of a multilayered chitosan gel in which each layer possessed different properties using a testing machine. The mechanical properties were evaluated for each layer.^52^ Nguyen et al. also examined the compressive modulus of multilayered constructs of poly(ethylene glycol) (PEG)-based hydrogel which exhibited spatially varying mechanical properties.^53^ They characterized each individual layer by separating each layer within the gel construct. The properties of each layer were measured independently. Here, we have developed new rheological methods that allow the characterization of not only the mechanical properties of individual layers within a 3D-printed gel but also the contribution of each layer to the resulting multilayered system. We show not only the importance of evaluating the mechanical properties of the gels after printing but also how using different protocols for rheological characterization could interfere on the determined rheological properties.

## Materials and Methods

### Materials

Fluorenylmethoxycarbonyl-diphenylalanine (FmocFF) was prepared as described previously.^54^ Dimethyl sulfoxide (DMSO) was purchased from Fisher Scientific and used as received. Deionized water was used throughout.

### Preparation of a Single-Layer Hydrogel

To prepare a gel using the solvent trigger approach, FmocFF is first dissolved in a suitable water-miscible organic solvent and then deionized water is added in one aliquot, which will lower the solubility of the molecule, thus forcing the self-assembly into one-dimensional (1D) structures. Here, we used DMSO as the solvent, such as the final DMSO/H_2_O ratio was 3:7. Typically, to prepare 1 mL gel of FmocFF 5 mg mL^–1^, 5 mg of FmocFF is dissolved in 300 μL of DMSO and pipetted into the container in which the gel is going to be formed, followed by the addition of 700 μL of H_2_O in one aliquot using a pipette. In the same way, to form 1 mL gel at a concentration of 15 mg mL^–1^ of FmocFF, 15 mg of the gelator is dissolved in 300 μL of DMSO followed by the addition of 700 μL of H_2_O in one aliquot using a pipette. The sample is then left overnight at room temperature, which ranged from 20 to 24 °C, without being disturbed, to allow gelation to occur. The sample is sealed with parafilm to avoid evaporation.

### Preparation of Multilayered Hydrogels

**Scheme 1:**
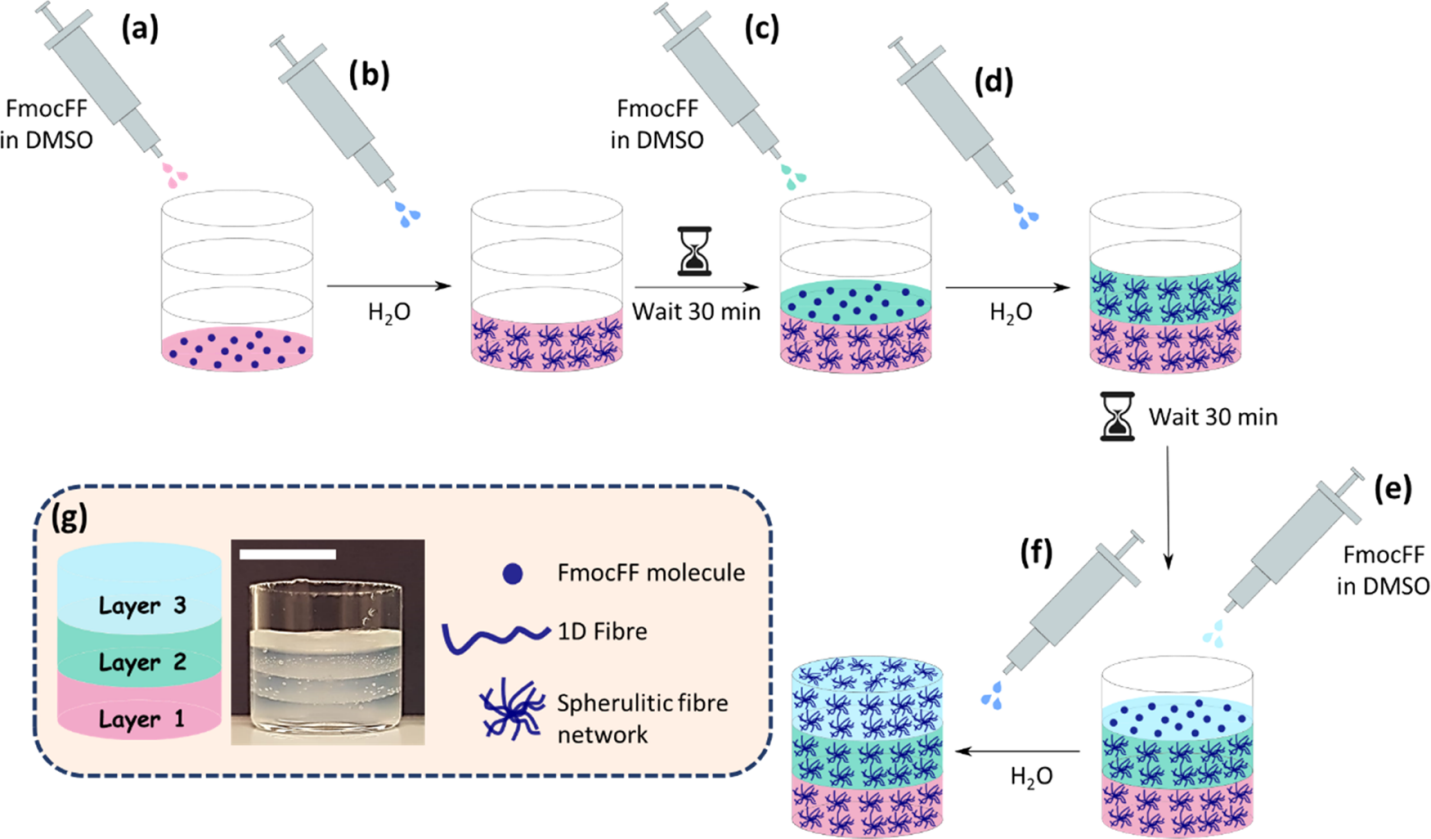
Schematic Representation of the Process by which a Three-Layer Hydrogel Is Formed In Situ Using a Layer-by-Layer Self-Assembly Method To form the first layer, (a) FmocFF dissolved in DMSO is pipetted into the container and (b) water is added to trigger gelation. Before starting to prepare the next layer, we wait 30 min to ensure the gel is completely formed. The same methodology was used to form the second (c, d) and third (e, f) layers. (g) From left to right, cartoon showing the resulting three-layered gel, a photograph of a three-layered hydrogel made of FmocFF 5 mg mL^–1^ using 30% DMSO, where each layer is 2.67 mm thick (the scale bar represents 1 cm) and cartoons describing the differences between an FmocFF molecule, fiber, and a spherulitic 3D network.

Multilayered hydrogels were prepared in situ. Multiple independent self-supporting layers of gels were formed one on top of each other using a solvent trigger. Specifically, we prepared three-layer hydrogels in situ. Three-layer hydrogels of multiple thicknesses were prepared as follows. First, a known amount of FmocFF dissolved in DMSO is pipetted into the container (Scheme 1a), followed by the addition of deionized water in one aliquot. Once the water is added, there is a phase separation where nucleation centers are formed, followed by the growth of fibers, that expand and form a spherulitic multidomain fiber network (Scheme 1b). Absorbance measurements at 600 nm were conducted for both FmocFF concentrations of 5 and 15 mg mL^–1^ to shed light on the assembly kinetics (Figure S11). For FmocFF at a concentration of 5 mg mL^–1^, there is an initial increase in absorbance as soon as the water is added, corresponding to the nucleation phase, followed by a gradual decrease in turbidity (Figure S11, cyan data). This change in turbidity is related to the formation of fibers underpinning the gel phase and a plateau is reached after 7 min. For FmocFF at a concentration of 15 mg mL^–1^, the same trend is observed where the system is initially highly scattering and then the turbidity decreases gradually, corresponding to the formation of fibers, until a plateau is reached after 10 min (Figure S11, pink data). The difference between the two concentrations is the final turbidity (higher turbidity for the more concentrated system) as well as the time needed for the assembly process to be completed, being 7 and 10 min for concentrations of 5 and 15 mg mL^–1^ of FmocFF, respectively. Out of an abundance of caution, we waited 30 min before preparing the next layer. After this time, the same procedure was repeated, i.e., a known amount of FmocFF dissolved in DMSO was pipetted carefully on top of the first layer (Scheme 1c). This step is quite arduous since we need to make sure that the DMSO solution containing the gelator is well distributed along the surface of the previous layer but also avoid interfering with that layer. To do this, we distribute drops of the FmocFF solution in DMSO on to different points of the previous layer being careful to not put a lot of pressure when pipetting, to avoid breaking the base layer. Once there is solution covering all of the surface of the previous gel layer, we pipette the water in one aliquot but again applying mild pressure (Scheme 1d). Prior to the addition of water, if the FmocFF solution in DMSO is not well distributed along the surface, it is probable that the gel will not fill the dimensions of the container in which it is being made. Since the gel takes a few minutes to form, immediately after the water is added, we can help the gel cover the whole area using a tiny pipette tip to drag the “sol-to-gel” system to the walls of the container and wait 30 min before starting to prepare the next layer. After this time, we prepared the third layer following the same methodology. FmocFF in solution was added to the top of the second layer carefully (Scheme 1e), followed by the addition of water (Scheme 1f). As a result, three self-supporting independent layers of gels are formed (Scheme 1g). In all cases, we always made sure the DMSO solution containing FmocFF was uniformly distributed along all gel surface, thus ensuring homogeneous gelation when water was added. Parafilm was used to prevent evaporation or drying.

### Oscillatory Rheology

Rheological measurements were performed using an MCR 301 rheometer (Anton Paar) and Rheoplus/32 v3.40 software. Different geometries were used, including a four-bladed vane in cup geometry (Figure S1a) and a parallel plate, PP 12.5 (Figure S1b). The vane and PP12.5 geometries used for rheological measurements are the ST10-4V-8.8/97.5-SN18190 and the PP12.5-SN50710 models, respectively, both from Anton Paar. As we intend to compare the rheological properties of multilayered systems prepared in situ with those delivered using an extrusion-based 3D printing technique, we designed and 3D-printed a container suitable for both techniques (Figure S1c).

Strain sweeps were carried out from 0.01 to 1000% strain at a frequency of 10 rad s^–1^ at a preset temperature of 25 °C. The linear viscoelastic region (LVER) was determined as the region, where *G*′ and *G*″ remain constant up to a strain amplitude at which the gel starts breaking (ca. 0.6–0.7%) and both moduli deviate from linearity. The values of *G*′ used throughout were taken as the average of the *G*′ values in the LVER. To define the critical strain (γ_c_), we draw a line tangent to LVER and another line tangent to the nonlinear region. The intersection of both lines will assert the value of γ_c_ (see Figure S2).

### Confocal Microscopy

Confocal images were taken using a Zeiss LSM 710 confocal microscope with an LD EC Epiplan NEUFLUAR 50× (0.55 DIC) objective. Samples before printing were prepared inside the 3 mL syringe with the tip cut. Once the gel was made, the plunger was used to expel the gel and a layer was cut off using a scalpel. For the gels after printing, they were premade as described for the samples before printing and then extruded using the optimized parameters onto a glass slide. Fluorescence from Nile Blue was excited using a 634 nm He–Ne laser and emission was detected between 650 and 710 nm. All gels triggered using a solvent switch were stained with a 0.1 wt % Nile Blue A solution in water. The Nile Blue was added to the DMSO–gelator solution to a final Nile Blue concentration of 2 μL mL^–1^ of gel.

### UV–Vis Absorption Spectroscopy

Absorbance spectra for FmocFF gels were collected over time (30 min) at 600 nm on an Agilent Cary 60 UV–vis spectrophotometer (Agilent Technology, California) using Cary WinUV, kinetic Application v5.0.0.999 software. All samples were prepared in a 1 mm path length quartz cuvette. First, water was added into the cuvette, and then a DMSO solution containing the gelator was added such that the final DMSO/water ratio was 3:7. After DMSO was added, the mixture was mixed quickly with the help of a needle. The total volume of gel examined was 300 μL for both concentrations of 5 and 15 mg mL^–1^ of FmocFF at 25 °C.

### Three-Dimensional Printing

The extrusion-based 3D printer used is a RepRap Ormerod 2 version 528.4 with some modifications. We have discussed it in detail in our previous publication.^33^ For the experiments, 3 mL single-layer hydrogels of FmocFF at a concentration of both 5 and 15 mg mL^–1^ were prepared in a 3 mL syringe by first adding the FmocFF dissolved in DMSO and then the water to trigger gelation. A long needle attached to a syringe was used to add the aliquots of FmocFF dissolved in DMSO and the water into the syringe. As explained in the Preparation of a Single-Layer Hydrogel section, we used DMSO as the solvent such as the final DMSO/H_2_O ratio was 3:7. Gels were left to gel overnight inside the syringes at room temperature and parafilm was used to seal the tip of the syringe to avoid evaporation of the solvent. Before printing, some parameters need to be optimized to achieve high-quality 3D-printed lines, among which we highlight the volume of the gel extruded, the speed of extrusion, the printer movement speed, and the printing height. For the different printing scenarios, each parameter was optimized to a rate of extrusion of 4 μL mm^–1^ and a shear rate of 1500 s^–1^. The shear rate refers to the rate at which the gel is extruded through the nozzle of the syringe and can be calculated from the following equation considering the pipe model , where *V* is the volume of gel extruded, *r* is the inner radius of the nozzle, and *t* is the time of extrusion. As an example, to print a 50 mm gel line at a shear rate of 1500 s^–1^ and rate of extrusion of 4 μL mm^–1^, 200 μL of gel were extruded in 0.13 s. The diameter of the nozzle used for extrusion was 2.2 mm. Then, we used the 3D printer to extrude our gels using the optimized parameters and they were left to settle for 5 min before being transferred into the rheometer for measurements.

Full characterization data and methods description are provided in the Supporting Information (SI).

## Results and Discussion

### Multilayered Gel Preparation

**Figure 1:**
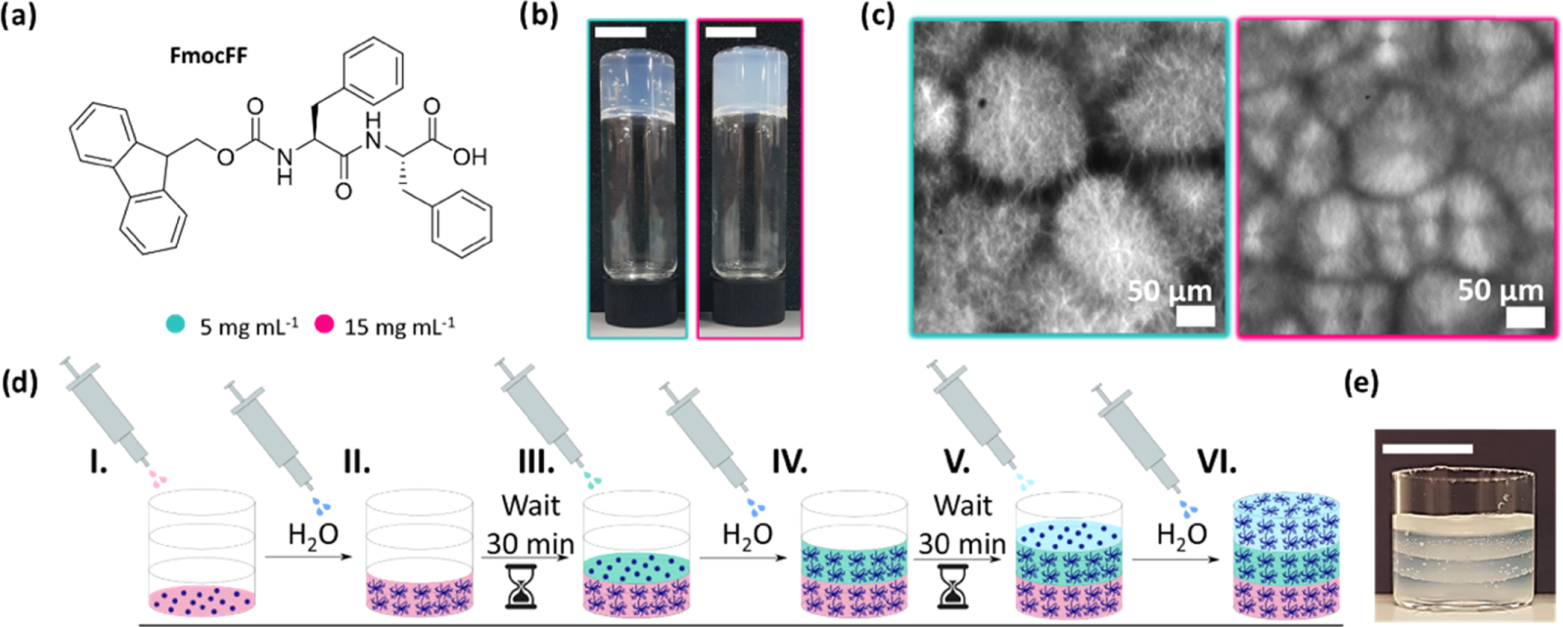
(a) Chemical structure of FmocFF. (b) Photographs of hydrogels prepared at a concentration of (left) 5 mg mL^–1^ and (right) 15 mg mL^–1^ of FmocFF using a DMSO/H_2_O ratio of 3:7. The scale bar represents 1 cm, and the gel volumes are 2 mL. (c) Confocal images of gels formed at concentrations of FmocFF of (left) 5 mg mL^–1^ and (right) 15 mg mL^–1^ at a DMSO/H_2_O ratio of 3:7. The scale bars represent 50 μm. (d) Schematic representation of the process by which a three-layered hydrogel is formed in situ. To form the first layer, (I) FmocFF is dissolved in DMSO is pipetted into the container and (II) water is added to trigger gelation. Before starting to prepare the next layer, we wait 30 min to ensure the gel is completely formed. The same methodology was used to form the second (III, IV) and third (V, VI) layers. (e) Photograph of a three-layered hydrogel where each layer was formed using FmocFF at a concentration of 5 mg mL^–1^ and 30% DMSO. Each layer is 2.67 mm thick (scale bar represents 1 cm).

FmocFF is one of the most widely used LMWGs as it forms gels at physiological pH and is commercially available (Figure 1a).^14,16,55^ FmocFF is an attractive low-molecular-weight gelator that forms stable gels at physiological pH, thus allowing for potential use in a range of biological applications including controlled drug delivery, cell culture, and tissue engineering.^14−16^ There are multiple methods to form gels from FmocFF, including a pH^17,18^ and solvent trigger.^19^ When using a solvent trigger approach, the molecule is dissolved in a water-miscible solvent, followed by the addition of water, which will lower the solubility and then self-assembly will occur. This usually drives a phase separation that results in spherulitic domains of fibers that entangle sufficiently to form a self-supporting gel.^20^

As a first step toward a comprehensive rheological characterization of 3D-printed LMWGs, multilayered hydrogels of FmocFF were prepared in situ (Scheme 1 and Figure 1d). This was achieved by preparing multiple independent self-supporting layers using a solvent trigger one on top of each other after the lower layer had gelled. For each layer, a known amount of FmocFF was dissolved in dimethyl sulfoxide (DMSO) and water added such that the final DMSO/H_2_O ratio was 3:7. This drives a phase separation that results in spherulitic-like domains of fibers being formed that entangle to form a self-supporting gel (Figure 1d).^56,57^ DMSO is broadly accepted below 10% (v/v) for biological purposes.^58^ However, since we intend to prove the applicability of rheological methods to characterize multilayered hydrogels, we considered DMSO at 30% (v/v) in view of well-defined FmocFF hydrogels being formed. The different hydrogel layers were prepared with different mechanical properties by varying the concentration of FmocFF. Specifically, we used two different concentrations of FmocFF, 5 and 15 mg mL^–1^ for the softer and stiffer gels respectively (Figure 1b). Gels made in both concentrations form similar microstructures with spherulitic-like domains of fibers (Figure 1c). We also examined the assembly kinetics for both concentrations of the gelator by measuring the changes in turbidity over time at 600 nm (Figure S11a). At this wavelength, FmocFF does not absorb light and therefore changes in absorbance can be ascribed to changes in turbidity. For FmocFF at a concentration of 5 mg mL^–1^, there is an initial increase in absorbance as soon as the water is added, corresponding to the nucleation phase, followed by a gradual decrease in turbidity, which corresponds to the formation of fibers.^56,57,59^ For FmocFF at a concentration of 15 mg mL^–1^, a similar trend can be observed for absorbance with the difference that the absorbance is much higher. In both cases, after 10 min there are no further changes in absorbance and therefore, we assume the gel network is totally formed. To ensure gelation was complete, we allowed 30 min to pass before preparing the next layer (Figure 1d).

In the following discussion, we initially focus on gels that are 8 mm thick in total. This thickness allows us to effectively demonstrate that we can probe and understand layered gels. We then move to gels of 2 mm total thickness, before finally comparing our data for layered gels to 3D-printed systems.

**Figure 2:**
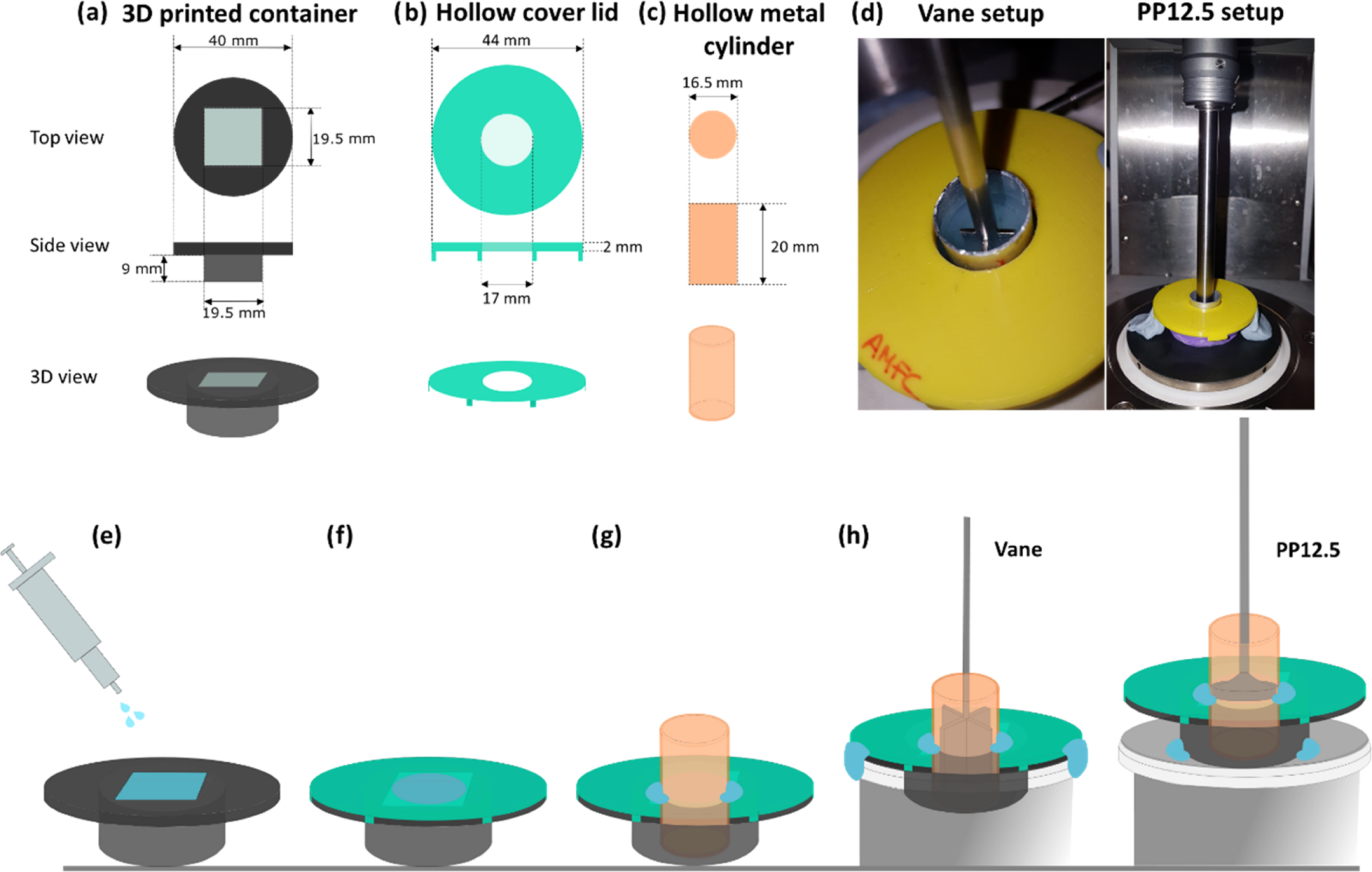
Layout of (a) the 3D-printed container, (b) the hollow cover lid, and (c) the hollow metal cylinder. (d) Photographs of (left) the setup for vane measurements and (right) PP12.5. (e–h) Schematic showing the procedure followed to load the samples for rheological measurements. (e) Gel is prepared inside the container; (f) then, a hollow cover lid is positioned on top of the container and (g) the hollow metal cylinder is inserted in the hollow and fixed in place with some Blu Tack; (h) then, we place it on the corresponding system depending on which geometry we will use and again some Blu Tack is used to ensure the container will not move during measurements.

Since we intend to compare the rheological properties of multilayered systems prepared in situ with those delivered using an extrusion-based 3D printing technique, we designed a specific container in which we prepare the gels that would be suitable for both techniques. We used a 3D-printed container (Figures 2a and S1c) which would allow us to directly extrude our gels using 3D printing and also prepare the same multilayered gels in situ. To probe these gels by rheology, we used two different measuring geometries, vane and parallel plate (PP) (Figures 2d and S1a,b). The PP geometry is widely used for hydrogel rheological characterization with sample thickness between 0.5 and 2 mm, while the vane is less common, but effective in conducting rheometry on soft materials that can be prepared in cups which could be susceptible to preshear caused by sample loading on to a plate.^60^ Both geometries measure bulk flow of material; however, their configuration is different in that a parallel plate measures from the top of the bulk sample, whereas the vane penetrates into sample without completely destroying the overall structure. We considered that the two different modes of operation could affect the resulting measured mechanical properties and trends associated with their layering. Furthermore, since the distance between the vane blades and the wall container in which the gel is made will affect the measured rheological properties (Figure S3), we optimized a setup that would allow us to minimize such distance. We used a hollow metal cylinder of 16.5 mm in diameter (Figure 2c), compared to the vane diameter of 7.5 mm, to “cut” the gel for measurements. As such, the amount of gel trapped between the vane blades and the metal hollow metal cylinder wall is minimum (4.5 mm), thus avoiding artifacts that could affect the stress applied to the bulk gel during measurements. We used the hollow metal cylinder setup for all measurements conducted using vane and PP geometries. Additionally, to ensure the concentric position of the hollow metal cylinder in the 3D-printed container, we used a cover lid (Figure 2b) for the container with a hollow in the middle in which the hollow metal cylinder fits perfectly.

Summarizing, the procedure used to prepare the multilayered gels for rheological measurements is as follows; first, we prepare the multilayered gels inside the 3D-printed container as explained previously (Figure 2a,e). Then, we settle the hollow cover lid on top of the container (Figure 2b,f) followed by the hollow metal cylinder (Figure 2c,g) to chop the gel and some Blu Tack to make sure it will not move during measurements. Finally, we place it into the corresponding rheometer system and set the corresponding measuring geometry (Figure 2d,h).

### 8 mm Multilayered Gel Systems Prepared In Situ

**Figure 3:**
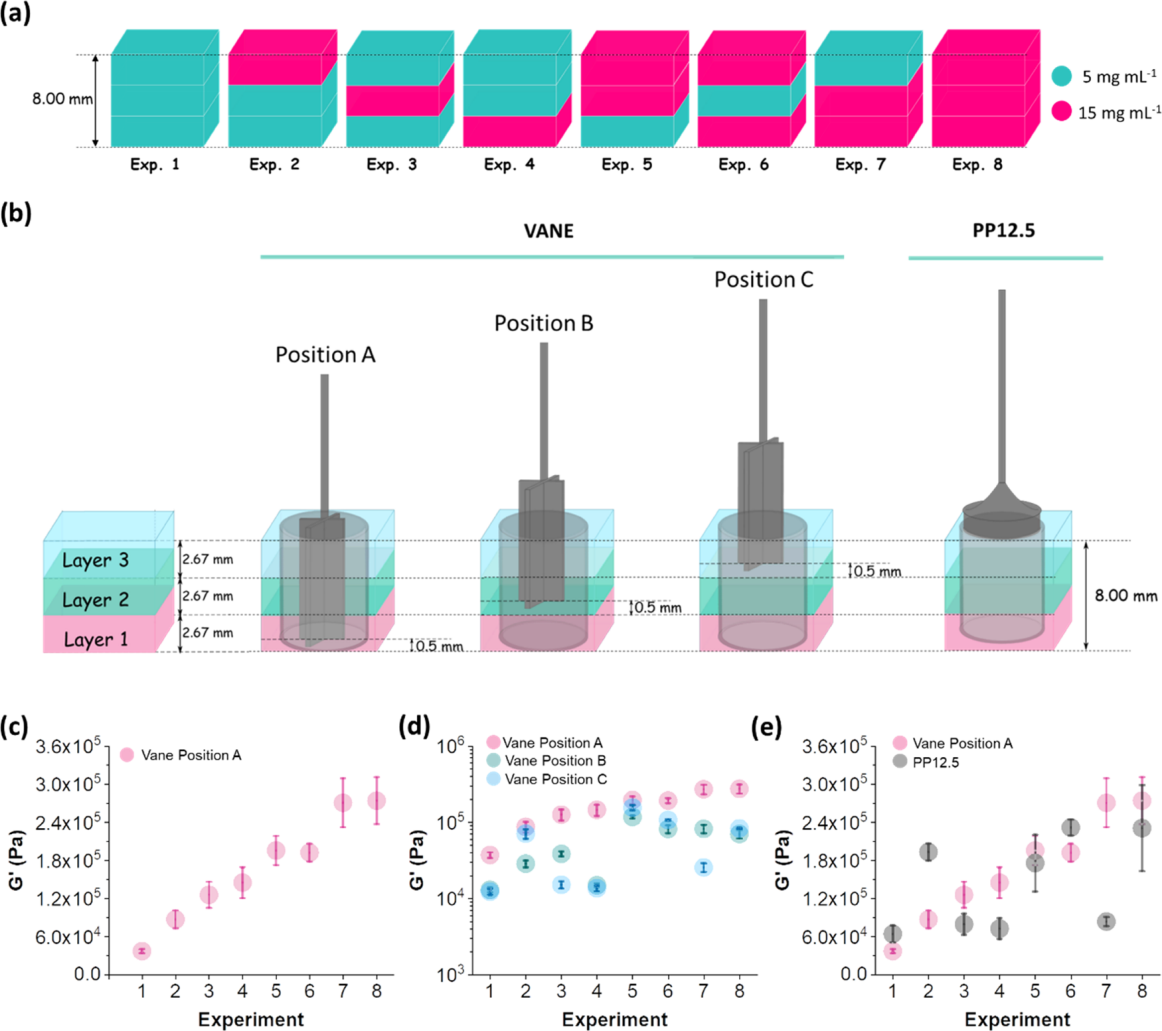
(a) Cartoon representing experiments 1–8, where each hydrogel is made of three layers. All cartoons represent 8 mm gels (2.67 mm each layer) in which the cyan and pink layers represent gels formed from FmocFF at a concentration of 5 and 15 mg mL^–1^ respectively. (b) Schematic representation of the different rheological protocols being used for the vane and PP12.5 geometries. Layers 1 (bottom), 2 (middle), and 3 (top) are represented in pink, green, and blue respectively. The vane geometry in positions A–C correspond to the vane embedded at 0.5 mm from the bottom of layers 1–3. The PP12.5 geometry is positioned on the surface of the top layer (blue) at an induced compressional normal force of 0.05 N. (c) *G*′ for experiments 1–8 using the vane in position A. (d) *G*′ values for experiments 1–8 in log scale using the vane in position A (pink circles), B (green circles), and C (blue circles). (e) Comparison of *G*′ for experiments 1–8 using both the vane in position A (pink circles) and the PP12.5 (gray circles). The error bars represent the standard deviation of three different samples of the same experiment.

Eight different experiments consisting of three-layered gel systems of 8 mm in thickness were carried out (experiments 1–8, Figure 3a). Together, they represent a gradient in the mechanical properties, which was modulated by changing the concentration and position of each layer within the gel system (Figure 3a).

Both vane and PP geometries were used to characterize experiments 1–8. PP geometry is not suitable for any samples with 8 mm thickness, but we use it to highlight the sensitivity of the vane. For measurements using the vane geometry, different positions of the vane were used. These are positions A–C, which correspond to the vane embedded at 0.5 mm from the bottom of layers 1–3 (Figure 3b). For the measurements carried out using the PP, normally, the geometry is manually lowered to the desired measuring gap. However, the measured stiffness can be affected by the induced compressional normal force during measurements.^61^ For gels formed using FmocFF, there is a dependence of the compressional force being applied to the gel before measurements on the resulting storage modulus (Figure S12a). As such, we used a setup where the PP geometry was lowered to a position where the detected normal force was 0.05 N. This force is low enough to detect the gel and stop the measuring system without compressing the gel significantly (Figure 3b).

The rheological properties of the hydrogels prepared using FmocFF were investigated by means of strain sweeps, using strains ranging from 0.01 to 1000% at an angular frequency of 10 rad s^–1^ (see Figures S13–S15, Supporting Information). First, experiments 1–8 were investigated using the vane geometry at positions A–C (Figures 3b and S13). The values for storage modulus, *G*′, were determined from the average of *G*′ in the linear viscoelastic region (LVER) for each experiment. The LVER was determined as the region where *G*′ and *G*″ remain constant up to a strain amplitude at which gel starts breaking (ca. 0.6–0.7%) and both moduli deviate from linearity (highlighted region in Figure S2).

For the rheological measurements using the vane in position A, where the vane is inserted in the bottom layer and touching all of layers 1–3, the stiffness increases linearly for experiments 1–8 (Figure 3c). Such a linear increase in *G*′ is a result of the specific distribution of the different layers within the gel system. Experiment 1 (three layers of 5 mg mL^–1^) and experiment 8 (three layers of 15 mg mL^–1^) are the controls, and in between them, the layered gels are made of different combinations of the two concentrations of FmocFF. If we consider experiments 2–4, the difference between them is the distribution of the layers (Figure 3a). Each of these is formed from two layers of a concentration of 5 mg mL^–1^ and one layer at a concentration of 15 mg mL^–1^, with the difference being the absolute position of the gel layer at a concentration of 15 mg mL^–1^. Considering the total concentration is kept constant (two layers of 5 mg mL^–1^ and one of 15 mg mL^–1^), we can interpret the increase in stiffness in experiments 2–4 as being due to the absolute position of the stiffer layer; there is an increase as the 15 mg mL^–1^ gel layer is closer to the bottom of the container where the vane is embedded. For experiments 5–7 again there is an increase in stiffness from 5 to 7. These gels are now formed from two layers of 15 mg mL^–1^ and one layer of 5 mg mL^–1^. Again, the stiffness depends on the relative positions of these layers, with the stiffest overall gel being that where both the 15 mg mL^–1^ layers are closer to the bottom (experiment 7). Notably, the stiffness for experiments 5 and 6 is constant. The difference between these is the distribution of the bottom and middle layers (one layer of 5 mg mL^–1^ and one of 15 mg mL^–1^). This is interesting since it reveals that both the bottom and middle layers contribute significantly to the total stiffness of the gel. But then a question arises: why do the stiffnesses for experiments 3 and 4 increase rather than staying constant as for experiments 5 and 6? We hypothesize that this has to do with the properties of the top layer, which is made of a concentration of 5 mg mL^–1^ for experiments 3 and 4, and 15 mg mL^–1^ for experiments 5 and 6. The stiffer layer (15 mg mL^–1^) is likely to dominate the vane measurements. As such, for experiments 3 and 4, the increase in stiffness is due to one 15 mg mL^–1^ layer being close to the bottom of the layered system, while for experiments 5 and 6 there are two layers of concentration 15 mg mL^–1^ that will dominate the stiffness measurements, minimizing the contribution of the 5 mg mL^–1^ layer. All of this shows that the rheological parameters being measured using the vane at position A are likely to be influenced by the properties of the neighboring layers in which the vane is inserted as well as the properties of each individual layer.

**Figure 4:**
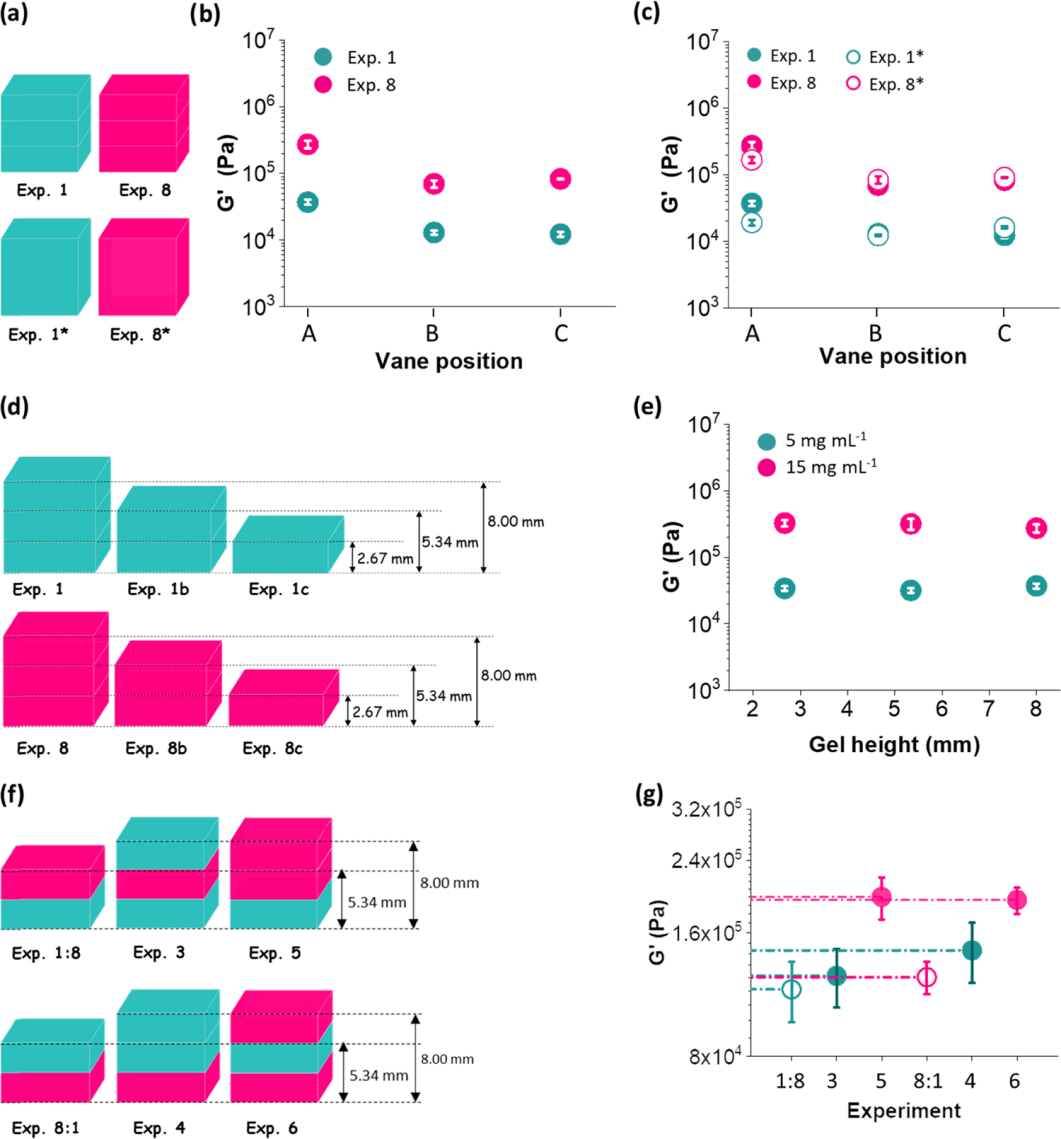
(a) Cartoons for experiments 1 and 8 (three-layered gels) and experiments 1* and 8* (monolayered gels). (b) *G*′ versus the vane at positions A, B, and C for experiments 1 (cyan circles) and 8 (pink circles). Vane positions A, B, and C correspond to a vane position of 0.5, 3.17, and 5.84 mm. The linear fits are represented with dotted lines and the trend is very similar for both experiments. (c) *G*′ versus the vane at positions A, B, and C for experiments 1 (filled cyan circles), 8 (filled pink circles), 1* (hollow cyan circles), and 8* (hollow pink circles). (d) Schematic representation of gels made of different heights by changing the number of layers. The cyan and pink layers represent gels formed from FmocFF at concentrations of 5 and 15 mg mL^–1^, respectively. (e) Evolution of *G*′ with gel height measured with the vane at position A. The linear fits are represented with dotted lines and are very similar for both concentrations. (f) Schematic representing different experiments made of different heights and distribution of layers. The cyan and pink layers represent gels formed from FmocFF at concentrations of 5 and 15 mg mL^–1^, respectively. (g) *G*′ values for the experiments represented in (f) using the vane in position A.

In the same way, we measured the rheological properties of layered gels 1–8 using the vane in position B (Figure 3d, green circles). Here, the vane is inserted into the middle layer (layer 2) so that it is only in contact with layers 2 and 3 (middle and top). In this case, the changes in *G*′ do not follow a linear trend as for the measurements of the vane in position A. There is an initial linear increase in stiffness between experiments 1, 2. and 3, but then the stiffness drops for experiment 4. For experiments 1 and 2, with the vane in position B, the increase in *G*′ is first due to the vane being in contact with two layers of 5 mg mL^–1^ and then one of the layers is swapped with a 15 mg mL^–1^. The increase in *G*′ between experiments 2 and 3 is due to the stiffer layer being closer to the layer in which the vane is embedded. Then, the *G*′ value drops for experiment 4, in which only two layers of 5 mg mL^–1^ are being measured. The values of *G*′ for experiments 1 and 4 are very similar, coinciding with the fact that in both experiments the two top layers are 5 mg mL^–1^. These results show the effectiveness of using the different positions of the vane to characterize different layers within a multilayered system. As another demonstration of the capability of this method, a decrease in stiffness when comparing experiments 5 and 6 is notable. The middle and top layers of experiment 5 are both made of gels at a concentration of 15 mg mL^–1^, whereas for experiment 6, these are at a concentration of 5 and 15 mg mL^–1^, respectively. The decrease in *G*′ is due to the vane being embedded within the 5 mg mL^–1^ layer in experiment 6, making the stiffness of the system lower compared with experiment 5.

The rheological properties of experiments 1–8 were also assessed using the vane in position C (Figure 3d, blue circles), in which it is only embedded in the top layer of the multilayered hydrogel. In this case, we can see a different trend for *G*′, where the stiffest values are found for experiments 2, 5, 6, and 8, which are the experiments in which the top layer is at a concentration of 15 mg mL^–1^. If we compare experiments 5 and 6, there is a notable decrease in *G*′, which corresponds with the middle layer being swapped from 15 to 5 mg mL^–1^. As such, when the vane is used in position C, not only the top layer is contributing to the resulting rheological parameters but also the properties of the layer below.

It is important to highlight that one would expect the modulus of a multilayered gel prepared at a constant concentration of the gelator (for example experiments 1 and 8) to be the same at different vane positions since. Experiments 1 and 8 show very similar *G*′ values for the vane at positions B and C. However, the *G*′ values for experiments 1 and 8 when the vane is in position A are higher. These differences could be explained as an edge effect of having the vane close to the bottom of the container in which the samples are prepared. Therefore, the position of the vane at which you measure the gel properties can affect the resulting *G*′ values, but when comparing the data at different positions this method is sensitive enough to detect differences in the mechanical properties of each individual layer within the multilayered gel system.

From the discussion above, it is clear that this method is capable of characterizing not only individual layers within a multilayered hydrogel but also the contribution of the neighboring layers. This is important for tissue engineering and regenerative medicine applications, as it is recognized that cells “sense” the neighboring elastic environment, which influences intracellular processes.^62,63^ It could also be used to detect whether neighboring gels layers or even surfaces could have an effect on bulk properties of gels.

We also used the PP12.5 geometry to characterize layered gels 1–8 (Figure S14). It is common to find in the literature the rheological properties of hydrogels being measured using a PP measuring system.^59,61,64,65^

Both parallel plates and vanes are considered “relative” measuring systems as they do not have a constant shear rate throughout the measuring gap so a point needs to be selected to measure the shear rate. The operational software calculates the shear rate by multiplying the rotational speed by a conversion factor for shear rate (CSR) owing to a specific point on the geometry. These same factors are applied when presetting strain. We show the differences in the measured rheological properties for experiments 1–8 using the vane in position A and the PP12.5 (Figure 3e). It is important to understand here that as we are using two relative systems, we should not necessarily get the same *G*′ values for vane and plate when measuring the same material; however, we can compare the trends of the measuring systems. The trend of the measured *G*′ values differs between the data collected with vane and those collected with the PP12.5 measuring system. As an example, for experiment 2 the stiffness values are very different using both geometries. The measured *G*′ values using PP12.5 and the vane are ∼2 × 10^5^ and ∼8 × 10^4^ Pa, respectively. For this specific experiment, the top layer is formed at a concentration of 15 mg mL^–1^, while the bottom and middle layers are formed from gels at a concentration of 5 mg mL^–1^. For the PP12.5 geometry, the top layer has a greater contribution to the measured properties than the middle and bottom layers. For experiment 7, where the top layer is at a concentration of 5 mg mL^–1^ and the middle and bottom layers are at 15 mg mL^–1^, we encounter the opposite situation; the highest *G*′ corresponds to the vane geometry measurement. This again shows that the PP12.5 measurements are more likely to be dominated by the top layer, which in this case is the softer layer, and make the *G*′ value be lower compared to the *G*′ measured with the vane, which takes into account the three layers. Furthermore, the measurements carried out using the PP geometry seem to present larger error bars after the critical strain (Figures S14 and S15). This could be due to the fact that the PP is much more likely to slip quicker compared to the vane geometry.

From these rheological results, we are able to not only characterize single layers in a multilayered hydrogel but also quantify the input that the rest of the layers are making to the system as a whole. To further characterize such systems, we implemented various tests. In Figure 3d, we show the effect of using the vane at different positions within a set of experiments where the distribution of layers is arranged in such a way that the mechanical properties of each gel system can be tuned. For experiments 1 and 8, where the gel is made up of three layers of 5 and 15 mg mL^–1^, respectively, the stiffness of the gel is independent of the vane position (Figure 4a,b). However, as mentioned above, the *G*′ values at position A are affected by the vane being close to the bottom of the container in which the gel is prepared and therefore needs to be taken into account. We also considered 8 mm gels made of one layer at a concentration of both 5 and 15 mg mL^–1^ of FmocFF (experiment 1* and experiment 8* respectively, Figure 4a). We evaluated the rheological properties using the vane at positions A, B, and C (Figure 4c) and compared with the counterparts three-layered systems (experiments 1 and 8). If the layers were not well integrated and secured together, the interface between the layers could lead to delamination or slip of the gel system and interfere with the rheological properties. We show very similar trends for *G*′ measured with the vane at positions B and C for one-layered and three-layered gels (Figure 4c). However, *G*′ values are slightly affected at position A for the multilayered gel compared to the bulk gel. We attribute these changes in stiffness to the fact that for a bulk gel there is only one gel–air interface, whereas for a three-layer gel, there are three air–gel interfaces and some mixing between the different layers could lead to effects on the resulting mechanical properties being measured. Furthermore, considering that the rheological *G*′ values for one-layer and three-layer gels hardly differ, we assume no slippage happens between the different layers within a multilayered hydrogel (slipping at the interface would be clear in the strain sweep, for example). There must be jamming of spherulites or mixing between adjacent layers that make them to stick together.

In an attempt to describe the interface between two layers of gel, we used confocal microscopy to obtain images in stacks in the *Z* direction of a multilayer hydrogel. This technique allows us to image the gel structure in the micrometer scale and therefore it was difficult to track a change or transition in the microstructure of two contiguous gel layers. In addition, rheology just describes the properties of the bulk material. As such, depicting the interface between two layers in the nano/macroscales is difficult but a key point which we will be following up in the future.

Similarly, we also examined the effect of changing the height of the gel being measured, keeping the vane at position A (Figure 4d,e). For both concentrations of FmocFF, we found that the *G*′ being measured is independent of the total thickness of the gel as long as the concentration used to make the gel is constant.

In an attempt to find other factors that would affect the mechanical properties being measured, we considered two-layer gel systems made of a concentration of 5 and 15 mg mL^–1^ layers (experiments 1:8 and 8:1, Figure 4f). We compared the *G*′ values of experiment 1:8 with experiment 3 (where a third layer is added) and experiment 8:1 with experiment 4 (where a third layer is also added) (see Figure 4f). We observed slight differences in *G*′ values when comparing the two-layer gels to the same gel in which a third layer was added (Figure 4g). Furthermore, we demonstrated that the mechanical properties of the third layer also affect the mechanical properties being measured (Figure 4g). This can be seen when we compare experiment 3 with 5, and experiment 4 with 6, where the difference is the concentration used to make the top layer.

### 2 mm Multilayered Gel Systems Prepared In Situ

**Figure 5:**
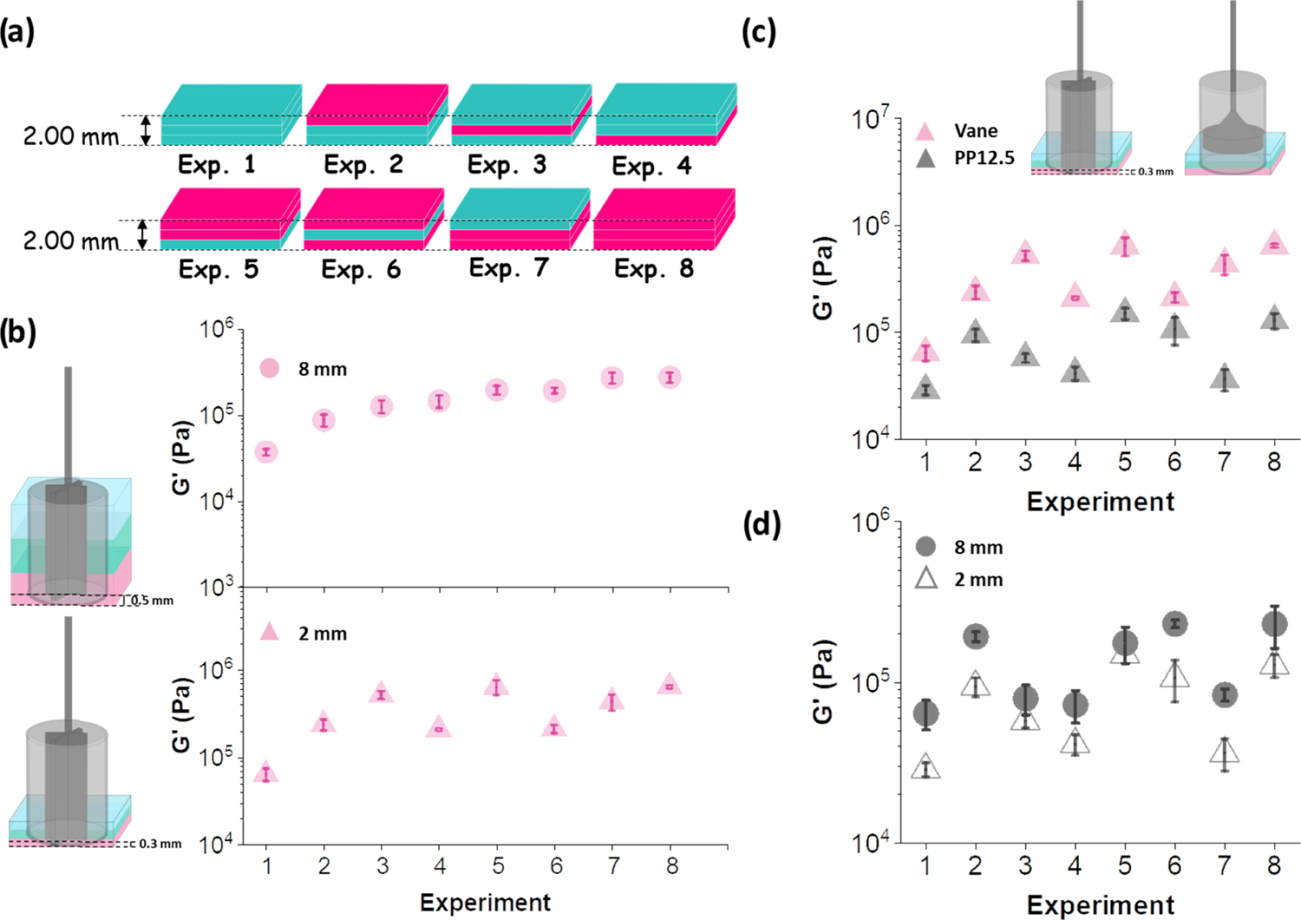
(a) Schematic showing experiments 1–8 where the height of the gel is 2 mm. Evolution of *G*′ for experiments 1–8 for (b) 8 mm (pink circles) and 2 mm (pink triangles) gels; (c) using vane at 0.3 mm from the bottom (pink triangles) and PP12.5 (gray triangles); and (d) using PP12.5 for 8 mm (gray circles) and 2 mm (gray triangles) gels. The error bars represent the standard deviation of three different measurements.

To date, we have shown a broad investigation of the rheological properties of 8 mm thick three-layered gels using the vane and PP geometries. For the vane measuring system, we have shown the relevance of the gel height on the rheological properties. For PP measurements, according to various standard testing methods, a gap of between 0.5 and 2 mm is recommended for obtaining reproducible data.^66^ At gaps larger than this it is difficult to know whether you are conducting a bulk measurement or just the contribution from the top of the material.^66^ As such, we decreased the height of layered gels 1–8 from 8 to 2 mm (Figure 5a). For three-layered 2 mm gels, each layer therefore now represents 0.67 mm of gel. For the vane measurements, we used a vane height of 0.3 mm to ensure the vane was touching at least the 50% of the bottom layer. For PP measurements, the same setup was used, in which the geometry compresses the gel 0.05 N before starting the experiment.

First, we examined the trend of *G*′ for experiments 1–8 in both 8 and 2 mm gel systems (Figure 5b). Individual strain sweeps of experiments 1–8 made of 2 mm in height and measured using the vane and PP geometries can be found in Figure S15. Interestingly, for vane measurements, there is sometimes a pronounced peak in *G*″ at the end of the linear viscoelastic region. Such a peak is often due to some microstructural rearrangement before the structure begins to yield and sometimes it can indicate a slow crossover into a slip regime. This behavior is not observed, or at least much weaker, in the 8 mm gel systems (Figure S14). Therefore, we propose this is due to heterogeneities at the layer boundaries. In the 2 mm gel systems, the range of layer borders is relatively larger compared to the overall sample thickness. The effect is also more pronounced with layers made of the higher concentration of the FmocFF gelator. As an example, if we compare experiments 1 and 8, there is a more pronounced peak of *G*″ for experiment 8 (Figure S15). It seems that with two layers of the higher concentration in connection to each other the *G*″-peak effect is the highest. On top of that, we highlight the slip effect observed in the PP measurements for 8 and 2 mm systems (Figures S14 and S15, respectively). The moduli decrease much faster toward increasing strain when using the PP12.5 system compared to the vane measurements.

As for 8 mm gels, the *G*′ values show an increasing trend for 2 mm gels, except for experiments 4 and 6, which deviate from the increasing trend (Figure 5b). This could be due to the vane not being entirely embedded in the bottom layer and therefore the greater contribution to the vane measurements relies on the middle and top layers. We examined the data for experiments 1–8 made on 2 mm gels. Initially, an increasing trend for experiments 1–3 is observed followed by a drop of the *G*′ value for experiment 4. From these results, one might contemplate the possibility of the vane not being in contact with enough of the bottom layer during measurements. However, if we observe the distributions of each layer for experiments 1 and 4, the only difference is the bottom layer being made of FmocFF at a concentration of 15 mg mL^–1^ for experiment 4, compared to a layer of 5 mg mL^–1^ of FmocFF for experiment 1. The values of *G*′ for both experiments 1 and 4 are different, experiment 4 being stiffer. With this, we confirm that the bottom layer is contributing to the rheological parameters being measured. Again, there is an increase in the value of *G*′ for experiment 5, this being higher than *G*′ for experiment 3 (this makes sense since the total gel concentration for experiment 5 is higher than for experiment 3), followed by a drop of stiffness for experiment 6, after which there is again an increase in *G*′ for experiments 7 and 8. We show that the bottom layer contributes to the measured *G*′ by observing the stiffness values for experiments 1 and 4. However, the deviation from the increasing trend of *G*′ observed for individual experiments, hints that although the bottom layer contributes to the measurements, the contribution is limited. Therefore, we examined the amount of gel from the bottom layer which is contributing to the measurements using the vane for 8 mm and 2 mm gels. Considering that for 8 mm gels (each layer consists of 2.67 mm of gel) the vane is used at a position of 0.5 mm, only 2.17 mm (∼81%) of layer 1 is contributing to the measurements. Conversely, for 2 mm gels (in which each layer represents 0.67 mm of gel) the vane is positioned at 0.3 mm from the bottom, this resulting in only 0.37 mm (∼55%) of layer 1 contributing to the rheological measurements.

We investigated further the differences in the rheological trends with the vane and PP12.5 (Figure 5c). When the multilayered gels display a gradient in concentration and are measured with a PP the values of *G*′ are heavily dominated by the top layer owing to the configuration of the geometry during the measurement. An important comparison in Figure 5c is between Exp 2 and Exp 7 for PP12.5. Exp 2 has one 15 mg mL^–1^ layer at the top and two layers of 5 mg mL^–1^ below, whereas Exp 7 has one layer of 5 mg mL^–1^ on the top and two 15 mg mL^–1^ below. The *G*′ value is higher in Exp 2 than Exp 7 even though the overall concentration in all three layers is actually higher in Exp 7. The vane does in fact record a higher *G*′ in Exp 7 than it does in Exp 2 because it is feeling the contribution of all three layers in the measurement. This really highlights the sensitivity of the vane compared to the parallel plate for multilayered systems, as we are not limited by measuring gap or contribution from the top layer. The thickness of samples that could be measured using a vane is only limited by the size of the vane itself and could be tailored for multilayers ranging from less than 2 mm up to over 50 mm for example.

Finally, comparing the rheological data for 8 and 2 mm gels using the PP12.5 (Figure 5d), we observe that the trends of *G*′ for experiments 1–8 are the same regardless of the total height of the multilayered gels; however, there are notable differences in the values of stiffness being measured for different gel thicknesses.

### Three-Dimensional-Printed Multilayered Gel Systems

Having proved the usability and effectiveness of the different rheological methods to characterize the mechanical properties of multilayered hydrogels, we move on to characterize 3D-printed systems using the vane and cup method we used above.

**Figure 6:**
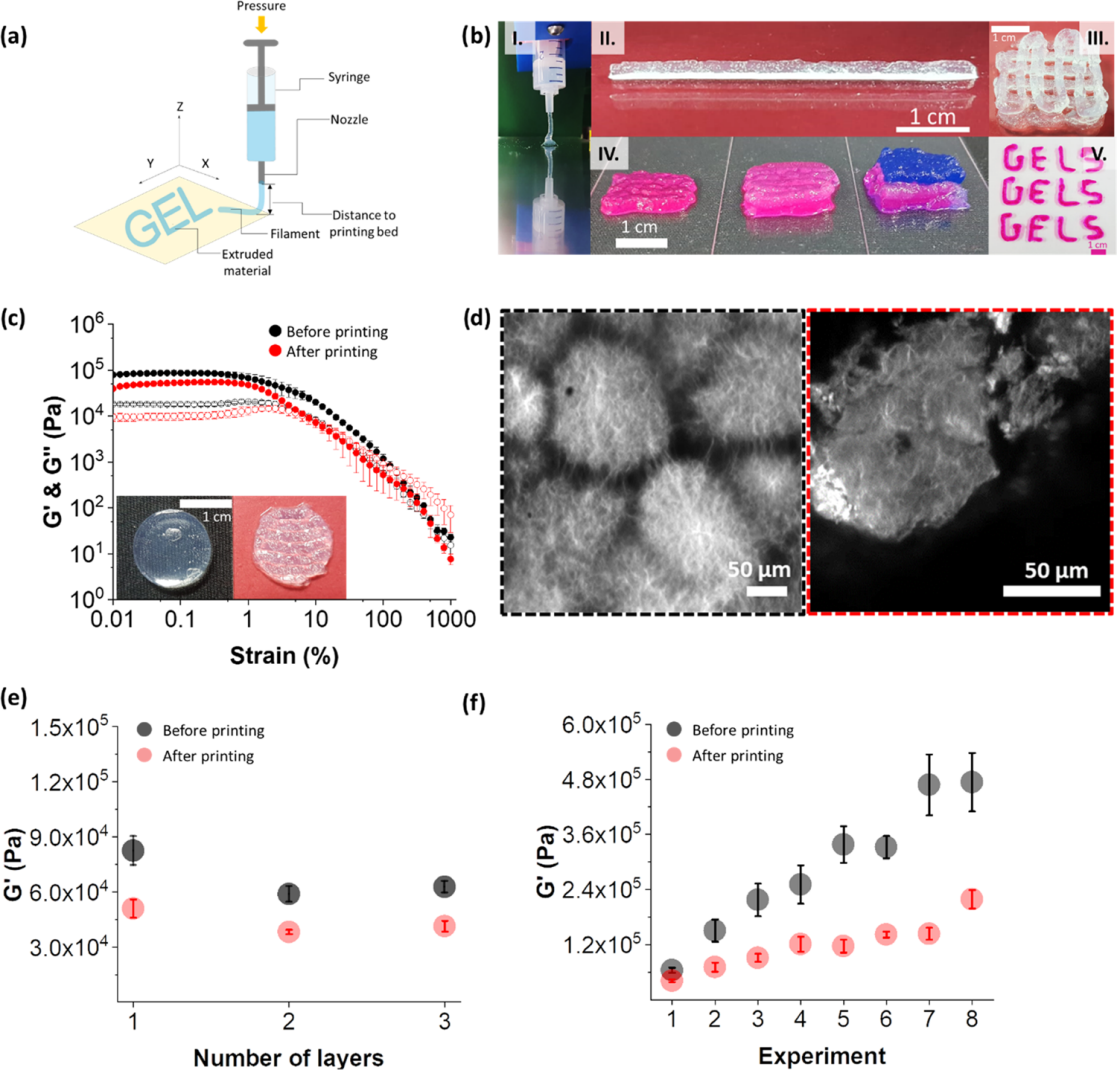
(a) Schematic diagram of an extrusion-based 3D printing setup. (b) Photographs of 3D-printed gels of FmocFF at a concentration of 5 mg mL^–1^. (I) Deposition of a gel filament onto the printing bed; (II) a 50 mm printed line; (III) scaffold of three printed layers; (IV) (left to right) one-layer, two-layer, and three-layer systems using a serpentine pattern and dyed with Rose Bengal (layer 1), no dye (layer 2), and Nile Blue A (layer 3); (V) printed text. All scale bars represent 1 cm. (c) Strain sweep for a single-layer FmocFF gel at a concentration of 5 mg mL^–1^ and 2 mm height before printing (black data) and one layer after printing (red data). The rheological measurements were carried out using the vane geometry at a measuring gap of 0.5 mm. The error bars represent the standard deviation for three measurements. The insets show photographs of a gel of FmocFF at concentration of 5 mg mL^–1^ (left) before and (right) after printing. (d) Confocal images for a gel of FmocFF at a concentration of 5 mg mL^–1^ (left) before and (right) after printing. The scale bars represent 50 μm. (e) *G*′ against the number of layers of gels made of FmocFF at a concentration of 5 mg mL^–1^ before (black data) and after (red data) printing. (f) Evolution of *G*′ for experiments 1–8 (black data) before and (red data) after printing. For all printed systems, a shear rate of 1500 s^–1^ and an extrusion volume of 4 μL mm^–1^ were used.

We have previously shown the suitability of some LMWGs for 3D printing^33^ and, therefore, gels with an underlying microstructure formed of spherulitic domains of fibers such as FmocFF can be printed effectively. Thus, lines of 50 mm length of FmocFF at a concentration of 5 and 15 mg mL^–1^ were printed and the printing parameters were optimized. First, FmocFF gels at a concentration of 5 mg mL^–1^ were extruded using a range of different shear rates (Figure S16a,b) while varying the extrusion volume from 4 μL mm^–1^ (Figure S16a) to 6 μL mm^–1^ (Figure S16b). Under visual inspection, lines printed at a shear rate of 1500 s^–1^ and a total volume of 200 μL (extrusion rate of 4 μL mm^–1^) exhibited smoother and more continuous printed lines (Figure 6b-II). The distance between the nozzle and the printing bed (Figure 6a) was also evaluated, 3 mm being the optimal height. In the same way, FmocFF gels at a concentration of 15 mg mL^–1^ were also evaluated under a range of shear rates for gels using a total volume of 200 μL per line. The same printing parameters used for the gels at a concentration of 5 mg mL^–1^ resulted in homogeneous printed gels at a concentration of 15 mg mL^–1^ (Figure S16c). We have previously shown the effectiveness of printing lines of FmocFF gels in multiple layers.^33^ Here, we also demonstrate that it is possible to print more complex structures made up of multiple layers of gel (Figure 6 b-III–V).

For such systems, many studies rely on the rheological characterization of the gels before printing due to the difficulty of doing so for gels post-extrusion. Here, we show the effect that extrusion-based 3D printing has on the properties of an FmocFF hydrogel. As a preliminary test, a single layer of FmocFF gel at a concentration of 5 mg mL^–1^ was extruded from a 3 mL syringe (Figure S16a) into a container (Figure S1c) in a serpentine pattern (Figure S18b) through the nozzle (inner diameter of 2.2 mm). The height of the printed gel was evaluated using the rheometer (more information is given in the SI, Section S1.1.7, p S12), and was found to be 1.86 ± 0.06 mm. Even if we showed above that differences in the height of the gel do not affect the rheological parameters being measured (Figure 4e), and either the microstructure of the gel being formed (see Figure S17), we prepared a gel of 2 mm height for comparison with the printed gel using a nozzle of 2.2 mm of inner diameter. The rheological data show that the stiffness of the gel is affected by the printing process (Figure 6c). The storage modulus, *G*′, for the gel before printing is (8.25 × 10^4^) ± (7.84 × 10^3^), whilst the *G*′ for the printed gel is (5.08 × 10^4^) ± (5.01 × 10^3^). The differences in stiffness for the gel before and after printing are meaningful, showing that only 62% of the initial *G*′ value is recovered and thus it cannot be assumed that gels are not affected by the printing process. The microstructure is also affected by the printing process (Figure 6d). Before printing, the gel shows the presence of spherulitic domains of fibers. After extrusion, there is still presence of fibers although the microstructure domain has been disturbed, resulting in a microstructure with increased domain size. During extrusion, the spherulitic domains are sheared as they go through the nozzle of the syringe. As a result, the fibers are displaced, thus changing the size of spherulitic domains. It is important to highlight that gels which present a spherulitic microstructure are easy to extrude since we avoid the effect of random orientations during flow, as it is the case of dense fibrous networks.^38^ Further, the spherulitic domains underlying the gel microstructure are complex in nature, which could lead to some differences in the printed microstructures when trying to replicate the same printed gel system. However, we show here the reproducibility in terms of storage modulus for the hydrogels containing spherulitic domains of fibers after extrusion, which emphasizes that even heterogeneities in the microstructure can still underpin gels with reproducible bulk properties in the printed hydrogel.

We also examined the recovery of the mechanical properties of FmocFF gels at the two concentrations by applying a high shear rate to disrupt the gels (Figure S19). Recovery tests were performed applying a variable stress at a frequency of 10 rad s^–1^ (within the viscoelastic region). Initially, 0.5% of strain was applied while monitoring *G*′ and *G*″ over 180 s. A strain of 300% was then applied for 60 s then stopped, and immediately a strain of 0.5% was again applied for an additional 180 s to monitor recovery. We repeated this cycle three times for FmocFF at concentrations of 5 and 15 mg mL^–1^ (Figure S19a). The gels recover up to 47.5 and 29.5% of their original value of *G*′ after the first high shear deformation for concentrations of 5 and 15 mg mL^–1^ respectively. However, the gels start to breakdown significantly in the successive cycles for both concentrations of FmocFF. Therefore, the gel of FmocFF at a concentration of 5 mg mL^–1^ was allowed a longer recovery time (Figure S19b). After 12 h of recovery, the gel recovers up to 76% of the original *G*′ value.

We then moved on to inspect more complex systems: two-layer and three-layer gels of FmocFF at a concentration of 5 mg mL^–1^ were investigated (Figure 6e,f). Again, the differences in stiffness for gels before and after printing are notable, the printed gels being less stiff compared with the gels before extrusion. Gels of one, two, and three layers recover up to 62, 65, and 84% of their initial *G*′ values, respectively (Figure 6e).

Additionally, we were able to evaluate the mechanical properties of the gels in experiments 1–8 after printing (Figure 6f). Gels 1–8 before printing were prepared in situ in the 3D-printed container as mentioned earlier in this paper. Each gel is made of three layers, each layer 2.67 mm high. The three-layer systems are thus made up of 8 mm of gel. To measure gels 1–8 after printing, each layer was extruded in a serpentine pattern into the 3D-printed containers with the appropriate concentration of FmocFF. The vane in position A was used to evaluate the rheological properties of the gels before and after printing. Once again, we show the effect of the printing process on the mechanical properties of the printed three-layered gels. As for gels prepared in situ, there is also an increasing trend of *G*′ for experiments 1–8 after they are extruded. There is an initial sharp linear increase in stiffness for experiments 1–4, then the *G*′ starts to level off for experiments 4–7 with a gradual increase in stiffness and then again a sharp increase for experiment 8.

## Conclusions

We have developed a rheological method to characterize the mechanical properties of multilayered hydrogels prepared in situ and post printing with a high degree of control. We prepared three-layered hydrogels with tunable mechanical properties in each layer by varying the concentration of the gelator FmocFF layer by layer. From rheological results, we show that not only a high degree of control of the mechanical properties of the individual layers within the multilayered constructs can be achieved but also that the contribution of each layer to the resulting combined properties being measured can be assessed. We also emphasize the differences of using different measuring systems and thickness of the prepared gels for rheological measurement, as it impacts highly on the resulting properties being measured, again highlighting the need to measure gel properties as close to the intended use as possible for accurate representation of the investigated systems.

The mechanical properties of the gels before and after 3D printing have also been examined. We show that the properties of FmocFF-printed gels are highly influenced by the extrusion process. This is important for biological applications, where an appropriate environment for cell growth is crucial to ensure appropriate cellular functions. We present this study as a guide for assessing the mechanical properties of 3D-printed gels and we hope it will aid in the characterization of new biomaterials made with cutting-edge technologies such as 3D printing.

## Supporting Information Available

Description of oscillatory rheology measurements; strain sweeps experiments; description of vane geometry; characterization of 8 and 2 mm multilayered hydrogels using oscillatory rheology; optimization of measurements using the vane geometry; description of parallel-plate geometry setup; limitations and data corrections; estimation of the real gap/height of the gel made inside 3D-printed container; methodology for preparing gels for rheological measurements using vane and PP12.5 measuring systems; turbidity measurements of FmocFF at concentrations of 5 and 15 mg mL^–1^; effect of the normal force applied to the gel using PP12.5 geometry during measurements; rheological data for 8 mm three-layered gels using the vane geometry; rheological data for 8 mm three-layered gels comparing the measurements with vane and PP12.5 geometries; rheological data for 2 mm three-layered gels comparing the measurements with vane and PP12.5 geometries; photographs of optimized 3D-printed lines of gels of FmocFF; confocal images of FmocFF gels at a concentration of 5 mg mL^–1^ made at different heights; photograph of the syringe used for 3D printing and cartoon showing the serpentine pattern used for gel 3D printing; and recovery tests (PDF)
