# Supplementary material for: Mechanical Characterization of Multilayered Hydrogels: A Rheological Study for 3D-Printed Systems
Source: Biomacromolecules. 2021 Mar 18;22(4):1625–38. doi: 10.1021/acs.biomac.1c00078 (PMC8045019; doi:10.1021/acs.biomac.1c00078)
Supplement: Supplementary file 1 — bm1c00078_si_001.pdf [file bm1c00078_si_001.pdf]

Mechanical characterization of multi-layered hydrogels: A rheological study for 3D printed systems

Ana M. Fuentes-Caparrós,[a] Zaloa Canales-Galarza,[a,b] Michael Barrow,[c] Bart Dietrich,[a] Jörg Läuger,[d] Markus Nemeth[d] Emily R. Draper[a] and Dave J. Adams[a,]*

a School of Chemistry, University of Glasgow, Glasgow G12 8QQ, U.K.

b Department of Chemical Engineering, Faculty of Sciences, University of Granada, 18071 Granada, Spain

c Anton Paar Ltd, St Albans, AL4 0LA, U.K.

d Anton Paar Germany, 73760 Ostfildern, Germany

Supporting Information

Table of Contents

Section 1. Experimental details S2

1.1. Oscillatory Rheology S2

1.1.1. Strain sweeps S2

1.1.2. Vane geometry S3

1.1.3. 8 mm multi-layered hydrogels S6

1.1.4. 2 mm multi-layered hydrogels S7

1.1.5. Optimization of vane measurements S8

1.1.6. PP12.5 geometry. S9

1.1.7. Real gap/height of the gel made inside 3D printed container S13

1.1.8. Methodology for preparing gels for rheological measurements using vane and PP12.5 measuring systems. S14

Section 2. Supplementary Figures S15

Section 3. References S20

Section 1. Experimental details

1.1. Oscillatory Rheology

Rheological measurements were performed using a MCR 301 rheometer (Anton Paar) and Rheoplus/32 v3.40 software. Different geometries were used, including a four-bladed vane in cup geometry (Figure S1a) and a parallel plate, PP 12.5 (Figure S1ab). As we intend to compare the rheological properties of multi-layered systems prepared in situ with those delivered using an extrusion-based 3D printing technique, we designed and 3D printed a container suitable for both techniques (Figure S1c).

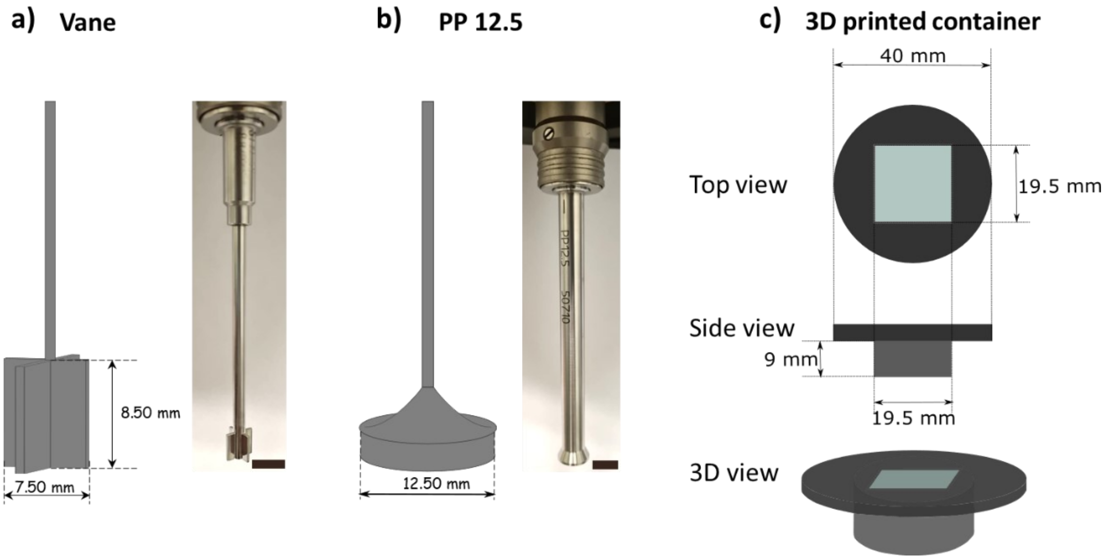

Figure S1. Diagrams and photograph of the different geometries used for rheological measurements and their dimensions; (a) cup and vane geometry, (b) parallel plate PP12.5 geometry. The scale bars represent 10 mm. (c) Diagram of the 3D printed container.

1.1.1. Strain sweeps

The strain sweeps were carried out from 0.01 % to 1000 % strain at a frequency of 10 rad s^{-1} at a pre-set temperature of 25°. The linear viscoelastic region (LVER) was determined as the region where G' and G" remain constant up to a strain amplitude at which the gel

starts breaking (~0.6-0.7 %) and both moduli deviate from linearity. The values of G' used throughout were taken as the average of the G' values in the LVER. To define the critical strain (γ_c), we draw a line tangent to LVER and another line tangent to the non-linear region. The intersection of both lines will assert the value of γ_c (see Figure S2).

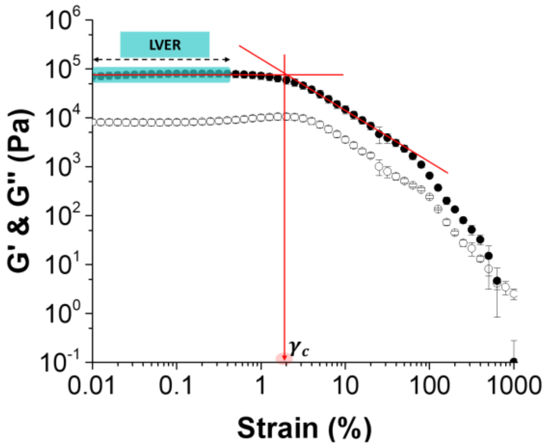

Figure S2. Example of strain sweep showing the LVER (highlighted in blue) and the γ_c (marked in red).

1.1.2. Vane geometry

The vane geometry is advantageous for measuring heterogeneous samples that may slip using the parallel plate.[1] However, some considerations need to be taken in order to get reliable measurements.

During measurements, the vane delineates a path along which the stress applied is inversely proportional to the square of the radius (Figure S3a-c).[2] Inside the limits defined by the vane blades radius (R_v), the material moves guided by the blades. However, the stress decays gradually outside these limits (Figure S3b). Differences in the radius between R_v and the wall container (R_c) will therefore have an effect on the calculated rheological parameters (Figure S3c). Similarly, the position of the vane during

measurements can affect the rheological properties being measured (Figure S3d). Differences in the vane position will determine the height/thickness of gel touching the vane blades as well as the distance from the vane to the bottom of the container. The rheological parameters could be influenced as a result of an edge effect of having the vane too close to the bottom of the container. Furthermore, when the vane is inserted within the gel sample, it drags down some of the gel as it passes through each gel layer. In Figure S3e we present a three layers gel system made of FmocFF at a concentration of 5 mg mL^{-1} and stained with Nile blue in either the top or middle layers respectively to better show the different layers (Figure S3eI,eII). When the vane is inserted within the multi-layered gel, damage to the gel through which the vane has been inserted can be seen, but there is little transfer for dyed gel (or dye alone) from one layer to the next (Figure S3eIII-IV). It is possible that as the vane passes through each layer, some gel is dragged down until the position where the vane is set for measurements. However, for a vane system the rheological parameters are calculated from the torque of the vane rotor as the blades are moving. Therefore, even if some of the gel is moved through to the bottom layer as the vane is inserted, we believe this will not interfere in the rheological parameters being calculated. To support this, when we compared the G' of single layer and multi-layered hydrogels we get very similar values, this showing that the gel mixing when the vane is inserted within the gel system does not affect the bulk material properties.

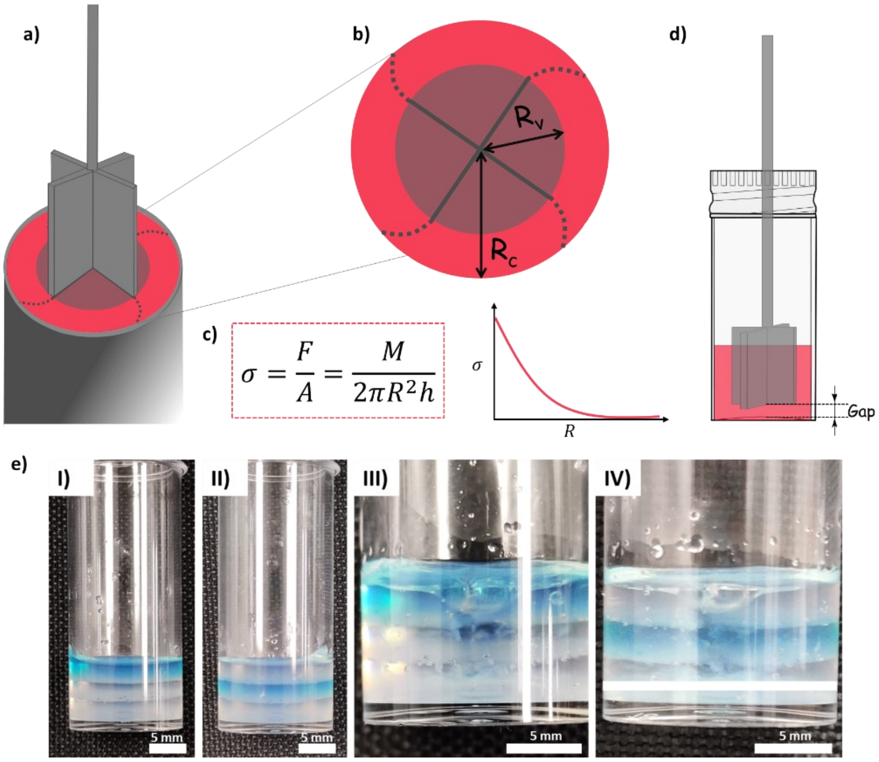

Figure S3. (a) Cartoon of a four bladed vane geometry inserted into a hydrogel (red) for measurement. (b) Inset of the shearing profile the material experiences during measurements. Inside the limits defined by the vane blade radius (R_v), the material moves guided by the blades. However, the stress decays gradually outside these limits; i.e., for the material trapped between the vane blades and the wall container. The distance between the center of the vane and the wall of the container is defined as Rc. (c) (Left) equation that defines the shear stress, where F is the force acting in shear direction (N), A is the surface (m^2), M is the torque (N m), R (or R_v) is the radius (m) and h is the thickness (m) of the material being examined and (right) profile showing the gradual decay of the shear stress as the radius increases. (d) Cartoon showing the position of the vane within a sample and the gap left between the vane and the bottom part of the container in which the gels are made. (e) Pictures of a three layers gel where each layer is stained with a different dye to better differentiate the different layers. (I, II) Side view of the gel before inserting the vane geometry, (III, IV) side views of the gel after we inserted and took out the vane geometry respectively. The three layers gel system was made of FmocFF at a concentration of 5 mg mL^{-1} and stained with Nile blue at either the top (I, III) or middle layer (II, IV).

On top of that, the vane geometry is a relative measuring system that do not have a constant shear rate throughout (like cones and cylinders) and therefore the CSR factor can be manually set by the user to calculate the speed at certain point in the measuring system. The CSS and CSR factors for the vane geometry used are 2700 Pa m^{-1} Nm^{-1} and 1 min s^{-1} respectively. The CSS factor on the geometry assumes that the vane rotor is fully submerged in the sample. Therefore, to calculate correct G' and G'' values we divided both moduli by the percentage of the measuring system (i.e., vane rotor) that was actually submerged in the multi-layered gel systems.

1.1.3. 8 mm multi-layered hydrogels

For 8 mm three-layered gels, each layer is 2.67 mm thick (Figure S4a). For these gel systems we developed different setups for the vane measurements; we varied the position of the vane in such a way that the distance between the bottom surface of each layer and the bottom part of the vane was kept constant. As a result, three different positions were used for measurements using the vane geometry; position A corresponds to the vane located at 0.5 mm above the bottom surface of layer 1, position B corresponds to the vane at 0.5 mm above the bottom surface of layer 2 and position C corresponds to the vane positioned at 0.5 mm above the bottom surface of layer 3 (Figure S4b).

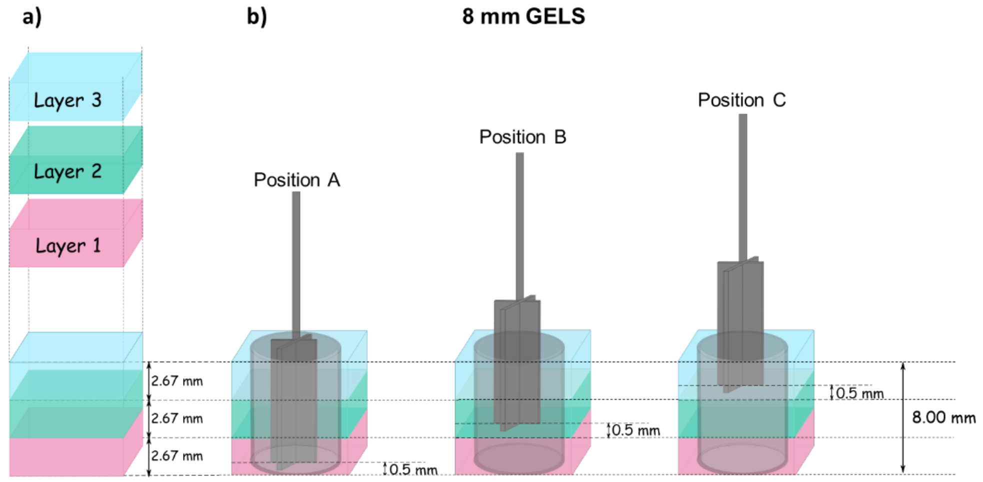

Figure S4. Schematic representation of 8 mm three-layered gels made in situ. (a) Illustration of a three-layered system in which each layer is made of 2.67 mm of gel, building up an 8 mm gel stack. (b) Schematic showing three different positions for the vane measurements; positions A, B and C correspond to the vane located at 0.5 mm above the bottom surface of layers 1, 2, and 3, respectively.

1.1.4. 2 mm multi-layered hydrogels

For 2 mm three-layered gels, each layer is 0.67 mm thick (Figure S5). In this case, the vane geometry was only used at one position; this is at 0.3 mm from the bottom surface of layer 1. Due to the thinness of each layer (0.67 mm), a gap of 0.3 mm was used in order to guarantee that the vane was embedded and in contact with at least 50% of layer 1, which would not be possible if a gap of 0.5 mm were used.

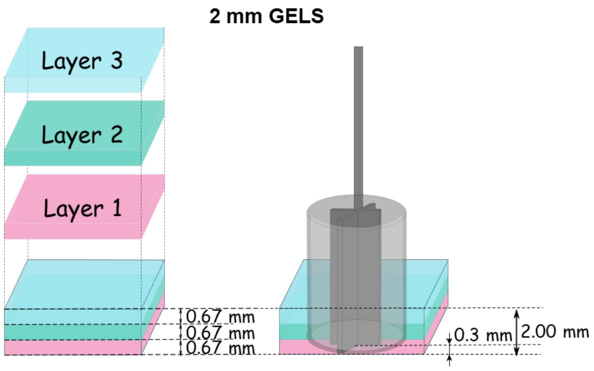

Figure S5. Schematic representation of 2 mm three-layered gels made in situ in which each layer is made of 0.67 mm of gel, building up a 2 mm gel stack. Also shown is the position of the vane 0.3 mm above the bottom surface of layer 1 for measurements.

1.1.5. Optimization of vane measurements

In order to get reliable data, it is important to control the position of the vane for each measurement, since every sample was prepared in a different 3D printed container. As such, we first evaluated what the 0 mm position would be for the vane in each container. In order to do so, we loaded each individual 3D printed container in the rheometer plate (Figure S6I-III) equipped with the vane and lowered the vane until a normal force of 0.5 – 0.6 N was detected by the vane (Figure S6IV). That position was identified as the 0 mm position, which corresponds to the vane touching the bottom surface of the container. We did this for every single container, since despite having been printed with the same design, the PLA plastic used to print them is firstly melted and then cooled down to room temperature during printing which causes slight differences in the dimensions between different 3D printed containers. Acetone vapor was also used to seal pores and smooth the container surface before first use. Once we optimized the setup for each individual 3D printed container, they were used independently for different samples and vane positions.

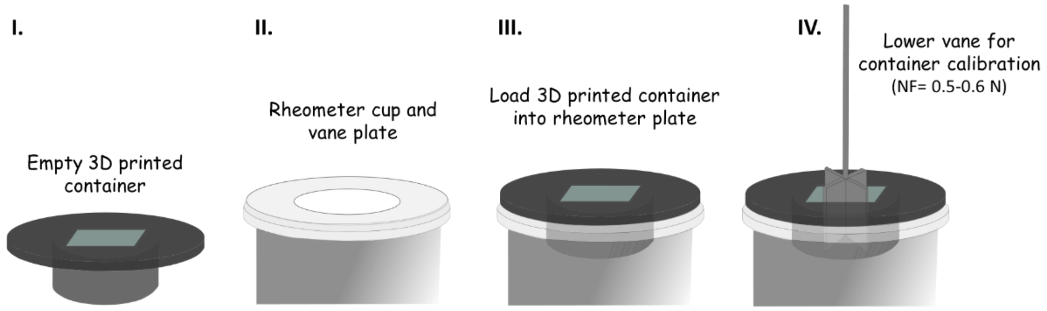

Figure S6. Schematic representing the process for calibrating every container that will be used to hold the multi-layered gels. (I), (II) and (III) loading of the 3D printed container into the rheometer plate and (IV) lowering of the vane until a normal force of 0.5-0.6 N is detected. This is set as the 0 mm position.

1.1.6. PP12.5 geometry.

The PP12.5 geometry used for rheological measurements is the PP12.5-SN50710 model (Anton Paar). When a PP geometry is used, the measuring gap can be controlled, which can be advantageous, as gels with different thicknesses can be measured. However, this has implications that need to be considered for measurements.

In a typical experiment, the sample is placed on the rheometer base plate and the PP geometry is lowered to the desired gap. Here, we used a controlled setup which lowers the PP12.5 geometry to a position where the detected normal force is 0.05 N (Figure S7a). That is, the PP12.5 will be in the measuring position when it just touches the surface of the gel and compresses it with 0.05 N of force.

During measurements, the shear rate is not constant along the geometry, being higher in the outer part of the PP (Figure S7a).[1] The shear stress applied also depends upon the size of PP used (Figure S7bII) and the thickness of the sample, i.e. the measuring gap (Figure S7bI).[3] To ensure homogeneous laminar flow of the material, the sample needs to range

between 0.5 mm and 2 mm in thickness. Laminar flow is not guaranteed if the sample height is outside of these limits. Likewise, the shear stress is also influenced by the size of the PP used, being higher for smaller sizes of PP geometries.[4] As for the vane geometry, the CSS (2617.89 Pa m^{-1} Nm^{-1}) and CSR (0.65364 min s^{-1}) factors were also manually set for the PP measuring system.

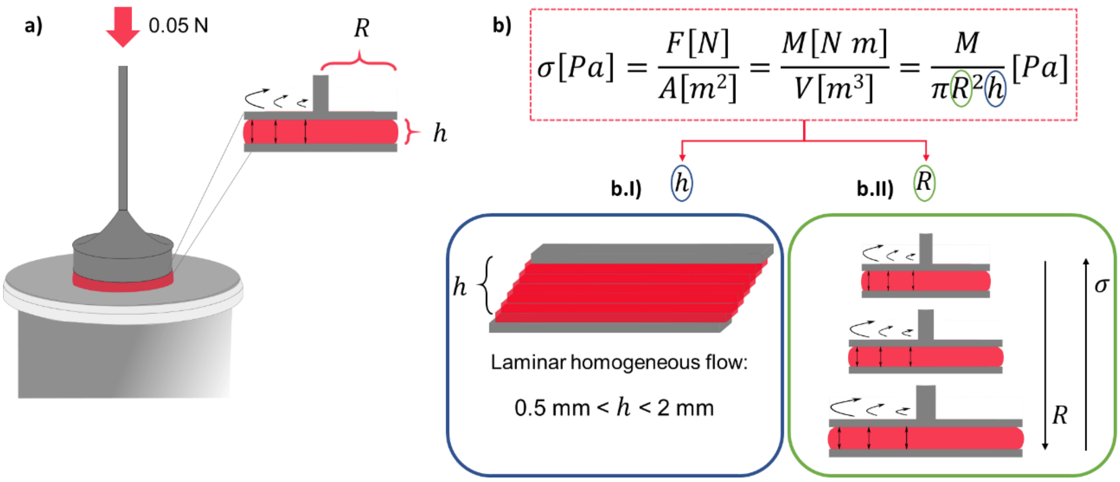

Figure S7. (a) Cartoon showing the setup of a typical experiment using a PP geometry, where the sample is placed between the geometry and the base plate. In this case, the PP12.5 geometry is lowered to a gap (h) where the detected normal force is 0.05 N. (b) Equation showing the dependence of shear stress (σ, in Pa) on the radius (R, in m) of the PP and the measuring gap width (h, in m); where F is the shear force (N), A the shear area (m^2), M the torque (N·m) and V is the volume of sample between the two plates (m^3). Schematic representing (b.I) the laminar flow of a material using PP and (b.II) the shear stress dependency on the size of the geometry used.

Multi-layered gels were prepared in the 3D printed containers in situ and using the extrusion-based 3D printing technique. The same experiments measured using the vane geometry were also analyzed with PP12.5. Three-layer gels of various thicknesses were prepared, building up 8 mm and 2 mm gel stacks.

For both 8 mm and 2 mm three-layered gels, the PP12.5 geometry is positioned on the top surface of the gel using the controlled setup where the PP stops when it detects a normal force of 0.05 N (Figure S8a,b).

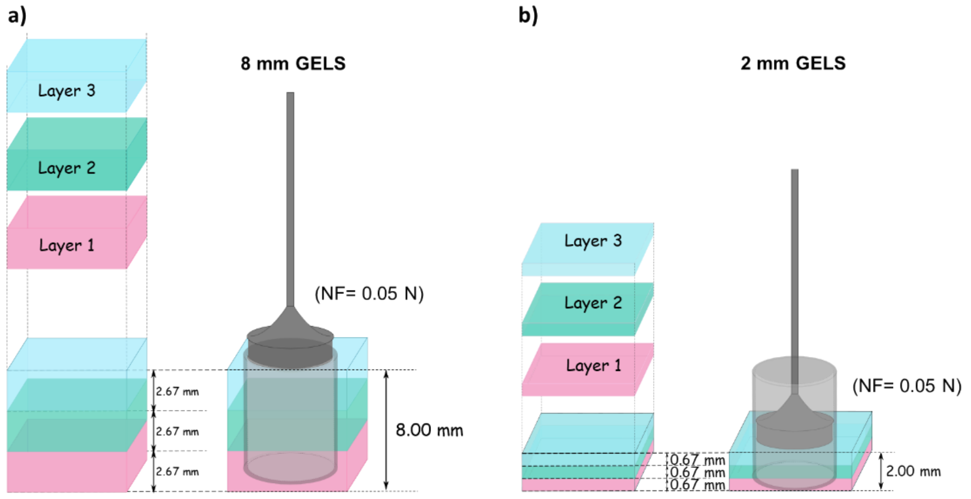

Figure S8. Schematic representation of (a) a three-layered system in which each layer is made of 2.67 mm of gel, building up an 8 mm stack and (b) a three-layered system in which each layer is made of 0.67 mm of gel, building up a 2 mm stack. In both cases a PP12.5 geometry is used for measurements. The PP12.5 is lowered using a controlled system that stops when the geometry detects a normal force of 0.05 N on the surface of the gel.

Before starting the measurements in a parallel system, setting the zero gap is necessary for the PP geometry. The zero gap is used as a reference for all gap settings. Likewise, setting the zero gap properly is fundamental since the rheometer will calculate the rheological parameter according to the zero-gap position. As an example, the shear strain γ is defined as $\gamma = \phi/h$, where ϕ corresponds to the deflection (in m) and h correlates the height/thickness/gap (in m) of the gel being measured. By definition, the strain depends upon the height of the gel being measured and hence it is important to appropriately set the zero gap. Normally, the zero gap is set on the base plate and then the sample is placed on top of it. However, in the present project the gels were made inside the 3D printed

containers. This would not allow to set the zero gap on the empty container before measurements since the gels were already prepared inside. Instead, we set zero gap on the base plate and we stuck the 3D printed container with the gel on top of it for measurements. Subsequently, the resulting strain values are based on the “measured gap” rather than the “real gap” (Figure S9a). As a consequence, we needed to apply a correction factor to rectify the values of strain. As an example, for a measured gap of 8 mm, a 100 % strain would equal to a deflection within the material of 8 mm. If instead of 8 mm, the real gap/height of the gel is 1 mm, the measured deflection of 8 mm would correspond to a strain of 800 %. In order to amend this, we applied a correction factor to the strain values measured. We firstly converted the deflection values from degrees to length units taking into account the perimeter of the circular PP12.5 geometry. Next, with the deflection values converted to distance, we worked out the real strain by dividing it by the height of gel (Figure S9b). The graph in Figure S9c, shows the differences in the strain profile before (black circles) and after (red circles) correcting the values of strain.

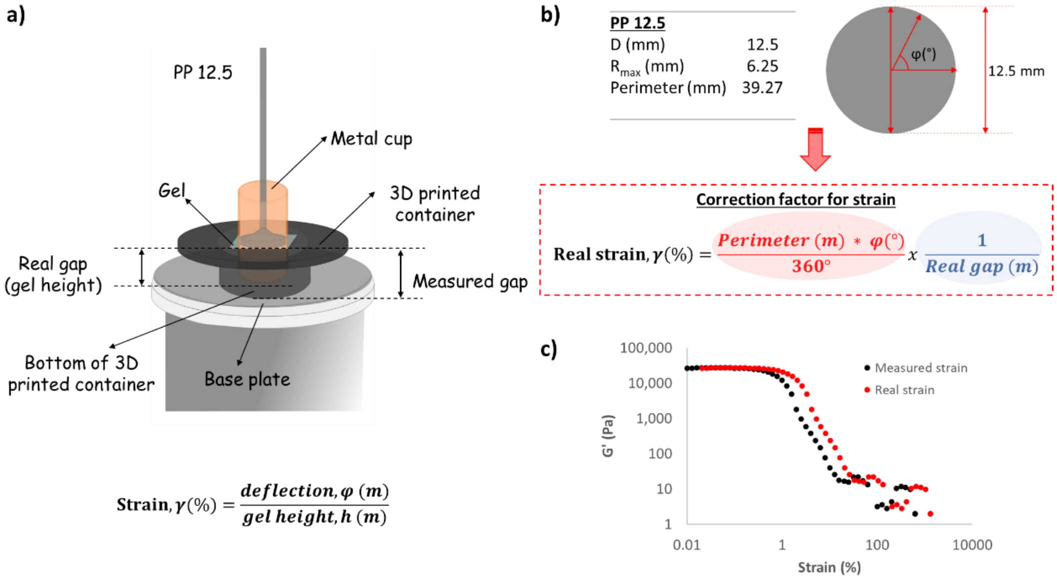

Figure S9. (a) Schematic representation of the setup used for rheological measurements using PP12.5 geometry on a gel made inside a 3D printed holder. The PP12.5 is on the top surface of the gel; the difference between this height and the bottom of the container is the real gap. (b) Correction factor applied to the measured strain values for a PP12.5 geometry. This factor takes into account the change in units of deflection from degrees to meters (highlighted in red) and also the height of the gel (highlighted in blue). (c) Graph representing one example of strain sweep comparing the rheological profile being measured by the rheometer (black data) and the modified profile taking into account the correction factor for strain values (red data).

1.1.7. Real gap/height of the gel made inside 3D printed container

The height of the gels was determined by subtraction of the measured gap with the gel made inside the container and the gap for the empty container (measuring the thickness of the container bottom). Both measurements were made using the controlled setup (0.05 N) for the PP12.5 geometry.

1.1.8. Methodology for preparing gels for rheological measurements using vane and PP12.5 measuring systems.

The procedure used to prepare the multi-layered gels for rheological measurements is as follows. Firstly, prepare the gel inside the 3D printed container as explained in Section 1 (Figure S10a,e). Then place the cover lid with a hollow on top of the container (Figure S10b,f) followed by the metal hollow cylinder (Figure S10c,g) to chop the gel into a repeatable shape and size and some Blu Tack® to make sure it will not move during measurements. Finally place the assembly into the rheometer and set the appropriate measuring geometry (Figure S10d,h).

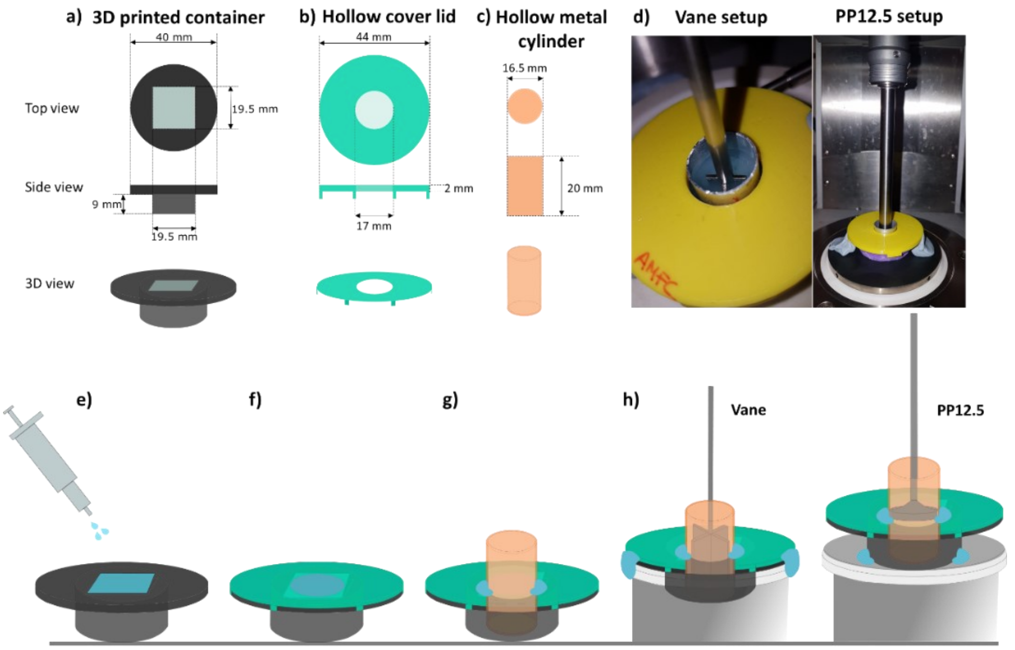

Figure S10. Dimensions of (a) the 3D printed container, (b) the cover lid and (c) the metal hollow cylinder. (d) Photographs of (left) the setup for vane measurements and (right) PP12.5. (e)-(h) Schematic showing the procedure followed to load the samples for rheological measurements; (e) Gel is prepared inside the container; (f) then a cover lid with a hole is positioned on top of the container and (g) the metal hollow cylinder is inserted in the hole and fixed in place with some Blu Tack®; (h) the entire assembly is then placed on the corresponding system depending on which geometry will be used and some Blu Tack® is used to ensure the container will not move during measurements.

Section 2. Supplementary Figures

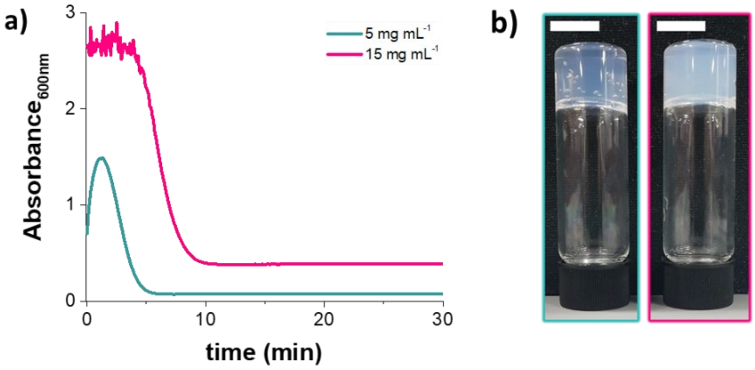

Figure S11. (a) Changes in absorbance at 600 nm with time after adding water to a solution of either FmocFF at 5 mg mL^{-1} (cyan) or 15 mg mL^{-1} (pink) in DMSO. (b) Photographs of gels of FmocFF at a concentration of (left) 5 mg mL^{-1} and (right) 15 mg mL^{-1}. Scale bars represent 1 cm.

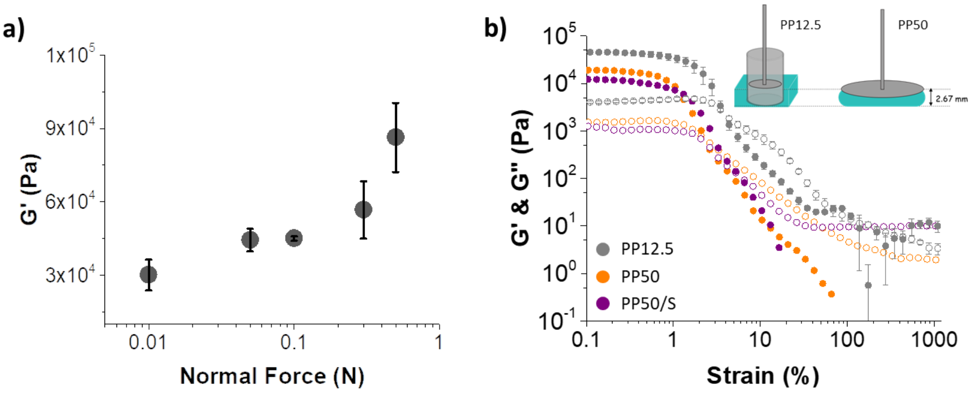

Figure S12. (a) Storage modulus G' versus the normal force applied to the gel before starting measurements using a PP12.5 measuring system on a 2.67 mm height gel of FmocFF at a concentration of 5 mg mL^{-1}. (b) Strain sweep for a 2.67 mm height gel made of FmocFF at a concentration of 5 mg mL^{-1} using PP12.5 (grey circles), PP50 (orange circles) and PP50/S (purple circles). The closed symbols represent G' and opened symbols refer to G''.

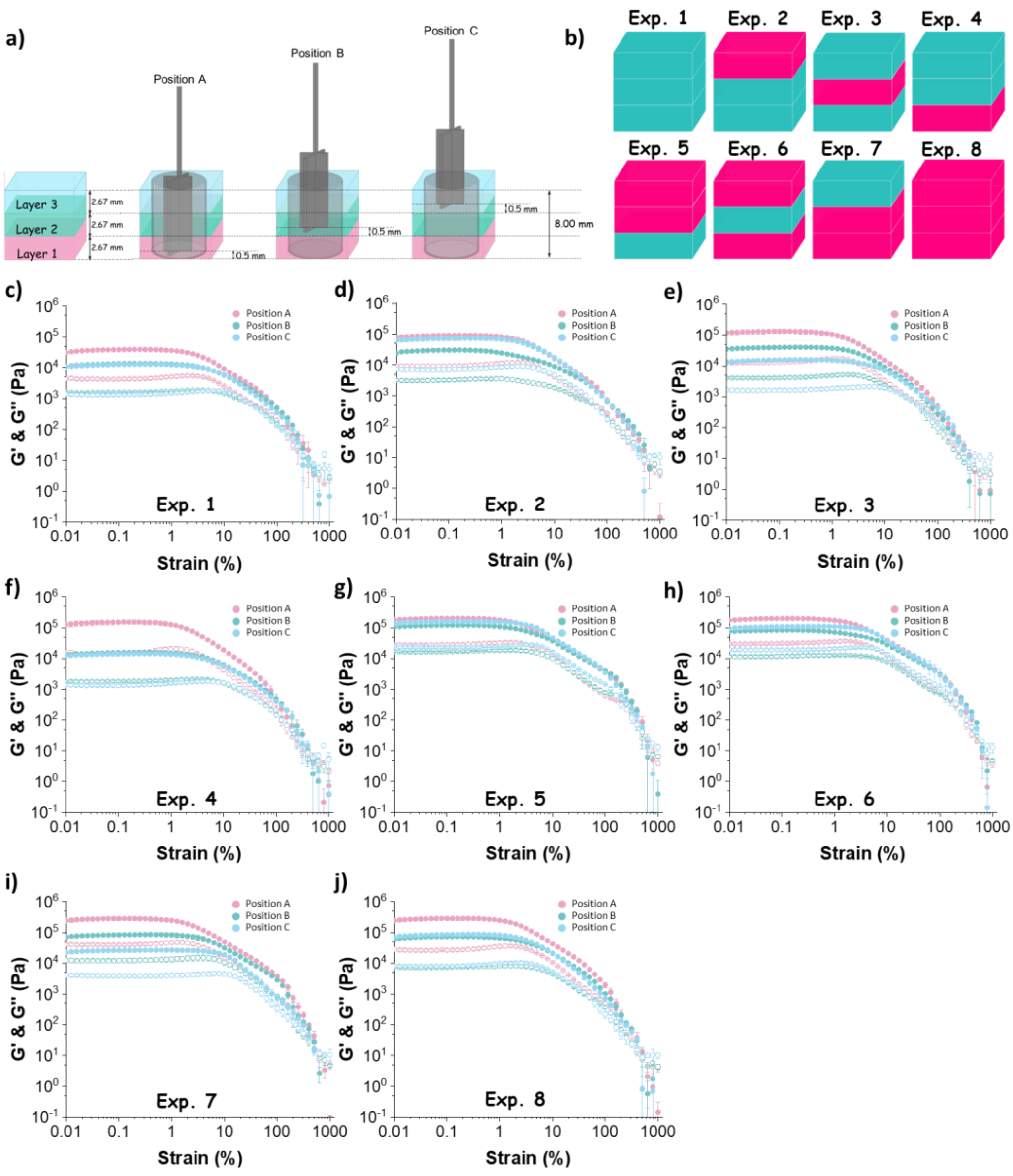

Figure S13. Rheological data for 8 mm three-layered gels using the vane geometry. (a) Schematic representing three different positions used for the vane measurements in an 8 mm three-layered gel; position A, B and C correspond to the vane located at 0.5 mm above the bottom surface of layers 1, 2 and 3 respectively. (b) Cartoons showing gel layers in experiments 1-8. Each gel stack is made up of 3 layers, where the cyan and deep pink layers represent 5 mg mL-1 and 15 mg mL-1 of FmocFF respectively. (c)-(j) Strain sweeps for experiments 1-8. The three sets of strain sweeps correspond to the vane measuring at position A (pink data), B (green data), and C (blue data). The error bars represent the standard deviation for three measurements. Closed and opened symbols represent G' and G'', respectively.

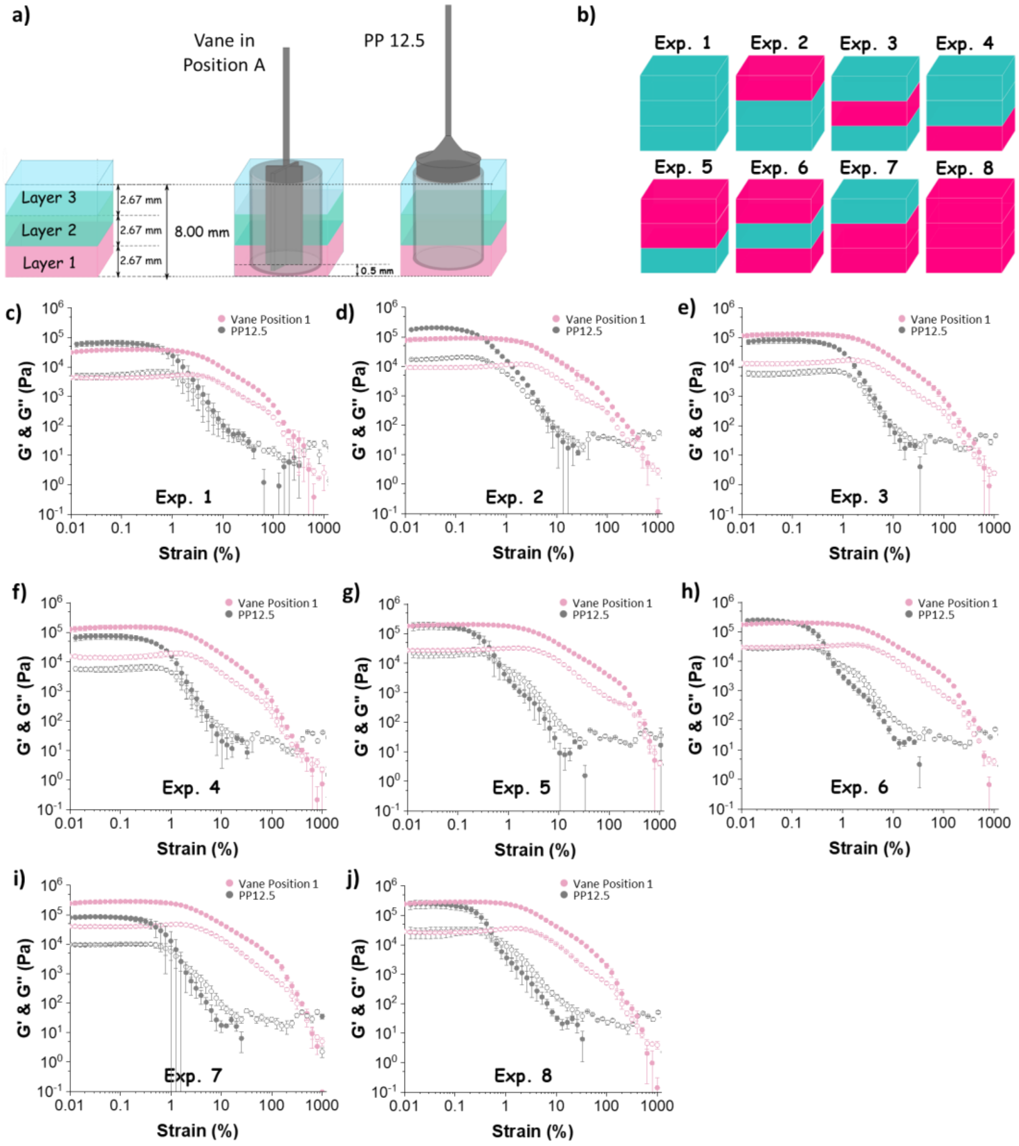

Figure S14. Rheological data for 8 mm three-layered gels comparing the measurements with vane and PP12.5 geometries. (a) Schematic showing the setup for the vane and PP12.5 measurements. (b) Cartoons representing experiments 1-8. Each experiment uses a stack of 3 gel layers, where the cyan and deep pink layers represent 5 mg mL^{-1} and 15 mg mL^{-1} of FmocFF respectively. (c)-(j) Strain sweeps for experiments 1-8. The pink data correspond to the measurements using the vane in position A and the grey data show the strain sweep measured using the PP12.5. The error bars represent the standard deviation for three measurements. Closed and opened symbols represent G' and G'', respectively.

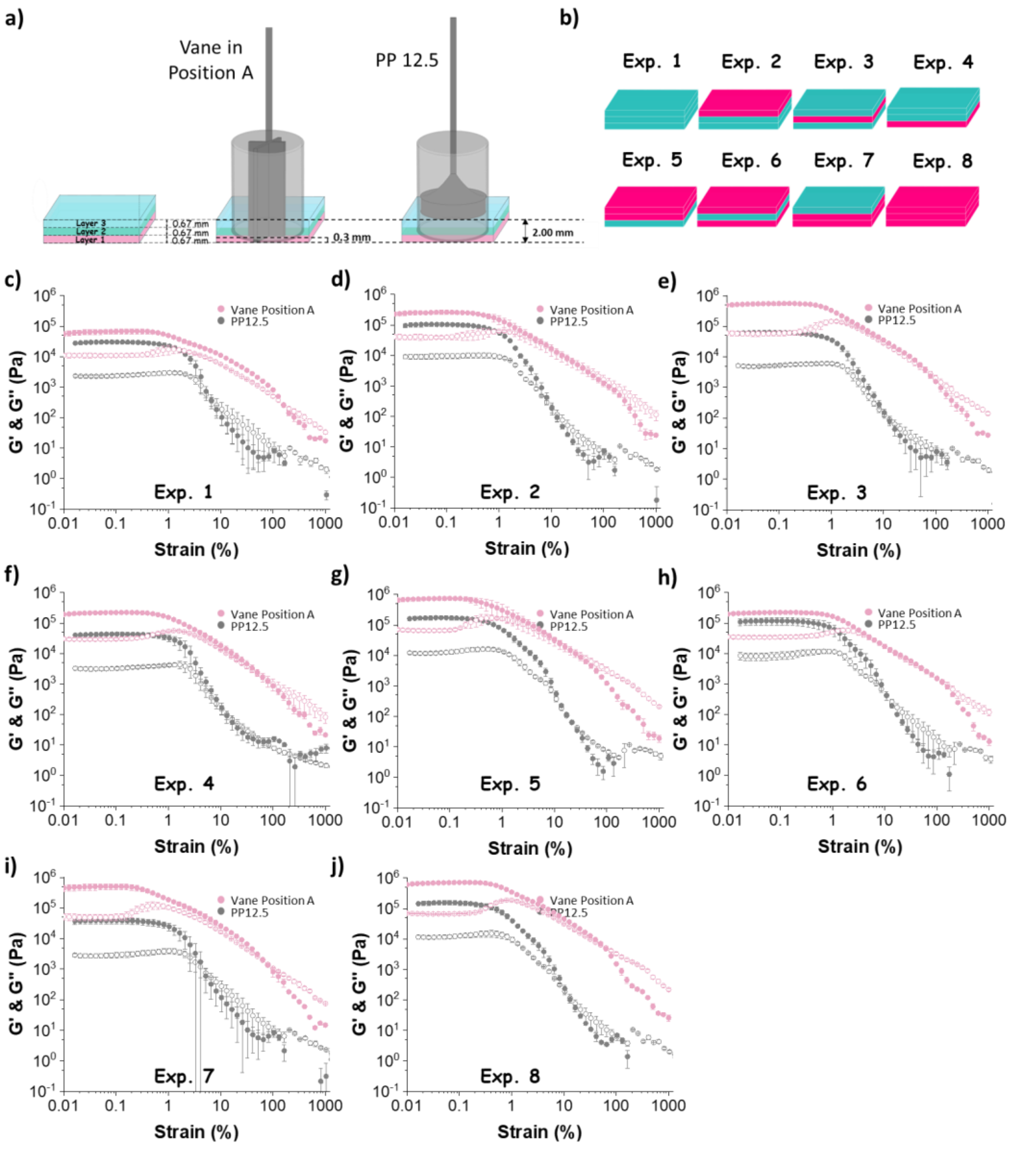

Figure S15. Rheological data for 2 mm three-layered gels comparing the measurements with vane and PP12.5 geometries. (a) Schematic showing the setup for the vane and PP12.5 measurements. (b) Cartoons representing experiments 1-8. Each experiment uses a stack of 3 gel layers, where the cyan and deep pink layers represent 5 mg mL^{-1} and 15 mg mL^{-1} of FmocFF respectively. (c)-(j) Strain sweeps for experiments 1-8. The pink data correspond to the measurements using the vane in position A and the grey data show the strain sweep measured using the PP12.5. The error bars represent the standard deviation for three measurements. Closed and opened symbols represent G' and G'' respectively.

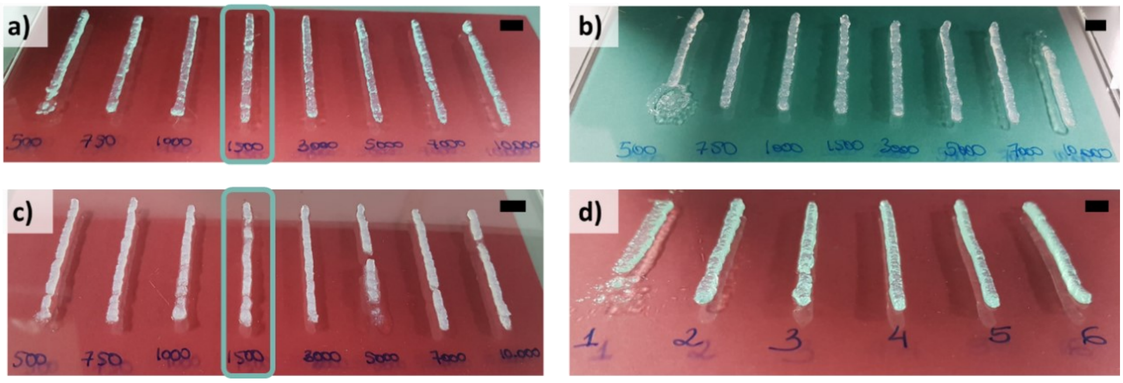

Figure S16. Photographs of optimized 3D printed lines of gels of FmocFF at a concentration of (a), (b) and (d) 5 mg mL^{-1} and (c) 15 mg mL^{-1}. In each photograph, the length of the printed lines is 50 mm. For (a), (b) and (c) the shear rate applied during extrusion is (from left to right) 500, 750, $1 \cdot 10^3$, $1.5 \cdot 10^3$, $3 \cdot 10^3$, $5 \cdot 10^3$, $7 \cdot 10^3$ and $1 \cdot 10^4$ s^{-1} and the distance between the nozzle and the printing bed is 3 mm. For (d) the shear rate applied is 1500 s^{-1} and each printed line was printed using a distance from the nozzle to the printing bed of (left to right) 1, 2, 3, 4, 5, and 6 mm. The total volume of printed gel is 200 μL for (a), (c) and (d), and 300 μL for (b). All gels were prepared in a 3 mL syringe in 3 mL volume before extrusion. The scale bars represent 1 cm in all cases.

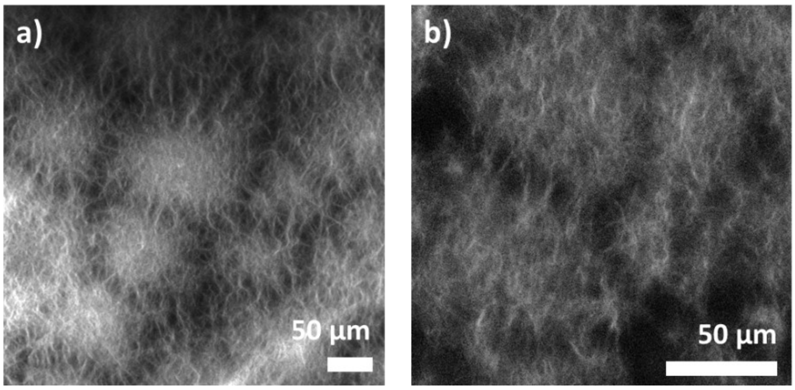

Figure S17. Confocal images of FmocFF gels at a concentration of 5 mg mL^{-1} and (a) 1 mm in height and (b) 2.67 mm in height. Gels were prepared in a container with the same dimensions as the 3D printed container used throughout (a photograph of the container used to prepare confocal samples can be found in Figure S18c). The scale bars represent 50 μm in both cases.

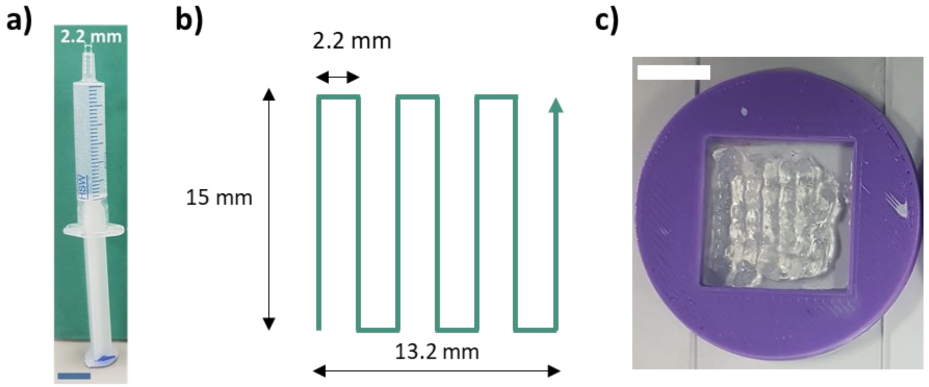

Figure S18. (a) Photograph of a 3 mL syringe with concentric slip tip. Inside it, 3 mL of gel at a concentration of 5 mg mL^{-1} was made. (b) Schematic showing the pattern used to 3D print the gels at different layers for rheological characterization. (c) Photograph of a single layer FmocFF at a concentration of 5 mg mL^{-1} gel printed using the serpentine pattern. The scale bar represents 1 cm in all cases.

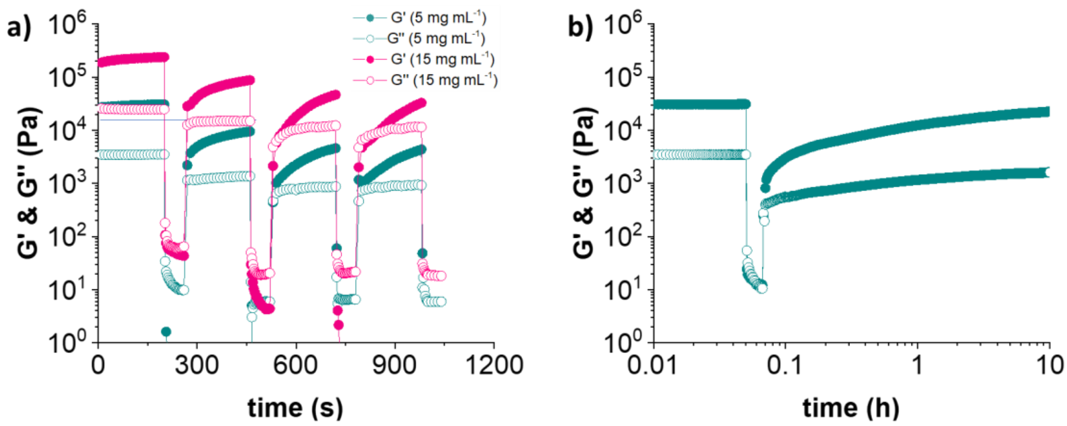

Figure S19. (a) Recovery test of FmocFF at concentrations of 5 mg mL^{-1} (cyan data) and 15 mg mL^{-1} (pink data). (b) Long recovery test for FmocFF gel at 5 mg mL^{-1}. Closed and opened symbols represent G' and G", respectively.

Section 3. References

1. Barnes, H. A., A handbook of elementary rheology. *University of Wales Institute of Non-Newtonian Fluid Mechanics* **2000**, 200.
2. Owens, C. E.; Hart, A. J.; McKinley, G. H., Improved rheometry of yield stress fluids using bespoke fractal 3D printed vanes. *J. Rheol.* **2020,** *64* (3), 643-662.

3. Perazzo, A.; Nunes, J. K.; Guido, S.; Stone, H. A., Flow-induced gelation of microfiber suspensions. *Proc Natl Acad Sci U S A* **2017,** *114* (41), E8557-E8564.
4. Macosko, C. W., *Rheology : principles, measurements, and applications*. VCH: New York, NY, 1994.
